# Exploring Sulfate as an Alternative Electron Acceptor: A Potential Strategy to Mitigate N_2_O Emissions in Upland Arable Soils

**DOI:** 10.1111/gcb.70428

**Published:** 2025-08-13

**Authors:** Hyun Ho Lee, Hanbeen Kim, Ye Lim Park, Marcus A. Horn, Jeongeun Kim, Jaehyun Lee, Sakae Toyoda, Jeongeun Yun, Hojeong Kang, Sang Yoon Kim, Jinho Ahn, Chang Oh Hong

**Affiliations:** ^1^ Institute for Microbiology Leibniz University Hannover Hannover Germany; ^2^ Department of Life Science and Environmental Biochemistry Pusan National University Miryang Republic of Korea; ^3^ Faculty of Land and Food Systems, the University of British Columbia Vancouver British Columbia Canada; ^4^ Department of Applied Environmental Science Kyung Hee University Yongin Republic of Korea; ^5^ School of Earth and Environmental Sciences Seoul National University Seoul Republic of Korea; ^6^ Climate and Environmental Research Institute Korea Institute of Science and Technology Seoul Republic of Korea; ^7^ Department of Environmental Chemistry and Engineering Tokyo Institute of Technology Yokohama Japan; ^8^ Research Center for Climate Sciences Pusan National University Busan Republic of Korea; ^9^ School of Civil and Environmental Engineering Yonsei University Seoul Republic of Korea; ^10^ Department of Agriculture Chemistry Sunchon National University Suncheon Republic of Korea

**Keywords:** Denitrification, Nitrous oxide, Sulfate reduction, Terminal electron acceptors, Upland arable soils

## Abstract

Agricultural activities are a significant source of nitrous oxide (N_2_O), accounting for approximately 60% of global emissions, highlighting the urgent need for innovative strategies to mitigate N_2_O emissions. Microbes conserve nearly as much energy with nitrate (NO_3_^−^) as oxygen (O_2_) respiration under limited O_2_ availability. Thus, microorganisms prioritize NO_3_^−^, limiting exploration of alternative electron acceptors (EAs) to inhibit N_2_O emissions through NO_3_^−^ respiration in upland arable soils. Current approaches remain insufficient, and the interactions between alternative EA reduction and pathways for N_2_O emissions remain poorly understood. This study evaluated oxidized iron, manganese, and sulfate as alternative EAs to reduce N_2_O emissions, along with the effects of zero‐valent metals (ZVMs). Metal sulfates (MSs) significantly minimized N_2_O emissions by inhibiting denitrification rather than altering nitrification in microcosms, as supported by isotope mapping and inorganic nitrogen concentrations. Among others, putative complete denitrifiers, N_2_O reducers, and sulfate reducers were stimulated, whereas ZVMs stimulated N_2_O emissions and 16S rRNA gene abundance. Moreover, the abundance of denitrifier‐related genes (*nirK*, *nirS*, *norB*, and *nosZ*) consistently decreased under MS treatments, while *dsrA* mRNA abundance significantly increased. Sulfate (SO_4_^2−^) addition reshaped the soil microbial community by enriching sulfur‐cycling taxa—including sulfate‐reducing and sulfur‐oxidizing bacteria—while suppressing nitrifiers such as *Nitrospira*, potentially disrupting nitrification–denitrification coupling. *Ureibacillus thermosphaerius*, harboring genes for denitrification and SO_4_^2−^ reduction, increased under MS treatment. These shifts likely redirected electron flow toward SO_4_^2−^ respiration, reducing NO_3_^−^ utilization and contributing to N_2_O mitigation. Field‐based manipulation experiments over 2 years demonstrated the feasibility of MSs in upland arable soils, reducing yield‐scaled N_2_O emissions by 21.5% without compromising crop yields. A systematic literature review and meta‐analysis revealed that SO_4_^2−^ application mitigated N_2_O emissions by an average of 9%, with over 70% of observations showing a decreasing trend, underscoring its potential as an effective soil amendment for sustainable agriculture.

## Graphical abstract

This study explored the use of sulfate‐based soil amendments as a new method to mitigate N_2_O—a powerful greenhouse gas—from agricultural soils. Laboratory and field experiments showed sulfate treatments significantly lowered N_2_O emissions without negatively affecting crop yields, mainly by altering soil microbial processes. These findings suggest sulfate amendments can be a practical and sustainable approach to reducing agriculture's climate impact.

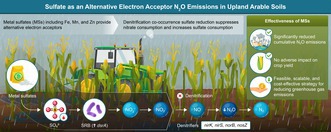

## 1 Introduction

Agricultural activities are a major source of nitrous oxide (N_2_O), contributing approximately 60% of global emissions (FAO 2021). As of 2025, agriculture emits an estimated 7.26 Tg N_2_O‐N y^−1^ globally, with 71.5% originating from arable soils treated with synthetic nitrogen fertilizers (NFs) (Reay et al. 2012). The nitrogen (N) cycle in agroecosystems is largely microbially driven and substantially influenced by the application of synthetic NFs, which stimulate microbial pathways responsible for N_2_O production (Davidson 2009; Fudjoe et al. 2023). Fertilized soils can convert up to 1.8% of applied N into N_2_O (IPCC 2019), generated directly through soil‐applied N and indirectly via pathways including nitrate (NO_3_^−^) leaching, ammonia (NH_3_) volatilization, and dissimilatory NO_3_^−^ reduction to NH_3_ (Ferland et al. 2024). Therefore, developing strategies that reduce N_2_O emissions while maintaining sufficient N availability for crops is critical to meeting the growing global food demand (Galloway et al. 2021; Mosier and Kroeze 2000). Technological advances such as slow‐release fertilizers (urea formaldehyde resin, polypeptide‐bonded urea), controlled‐release fertilizers, and the use of urease and nitrification inhibitors have shown promise in enhancing NF efficiency (Govil et al. 2024; Klimczyk et al. 2021; Zhang et al. 2024). In addition, advanced management practices, including split fertilization and deep soil injection, also offer benefits on N efficiency (Dong et al. 2022; Millar et al. 2010). However, there remains a need for innovative, scalable approaches that are not only agronomically effective but also economically feasible, labor‐efficient, and compatible with existing agricultural machinery.

Microbial‐mediated N processes in upland arable soils, primarily nitrification and denitrification, contribute significantly to global N_2_O emissions, accounting for approximately 70% of the total anthropogenic flux (Braker and Conrad 2011; Syakila and Kroeze 2011). Under typical aerobic conditions prevalent in well‐drained upland arable fields, nitrification, driven primarily by ammonia‐oxidizing archaea and bacteria as well as nitrite‐oxidizing bacteria, is generally the dominant N_2_O‐producing process during crop cultivation (Hu et al. 2022; Inatomi et al. 2019). Additionally, Wrage et al. (2004) highlighted nitrifier denitrification as an important contributor to N_2_O emissions under these aerobic conditions, further underscoring the complexity of the microbial pathways involved. However, denitrification, which typically requires anaerobic or substantially reduced conditions, can become a dominant pathway during episodic events such as heavy rainfall, irrigation, or temporary soil saturation. Under these transient anaerobic conditions, increased water‐filled pore space (WFPS) significantly promotes denitrification activity, leading to a short‐lived yet substantial peak in N_2_O emissions (Ruser et al. 2006; Wang et al. 2021). Consequently, despite the overall dominance of nitrification and nitrifier denitrification under common upland conditions, cumulative annual emissions can often be dominated by these episodic denitrification events (Ibraim et al. 2020; Scheer et al. 2020; Zhang et al. 2022). This duality in microbial contributions to N_2_O emissions underscores the need for tailored mitigation strategies, emphasizing interventions that specifically address denitrification dynamics under episodic and field‐relevant anaerobic conditions.

Soil microorganisms utilize terminal electron acceptors (EAs) in a thermodynamically determined sequence: oxygen (O_2_), then NO_3_^−^, followed by ferric iron (Fe^3+^), manganese (Mn^4+^), sulfate (SO_4_^2−^), and carbon dioxide (CO_2_) (Meulepas et al. 2010; Sivan et al. 2016). Among these, Fe^3+^, Mn^4+^, and SO_4_^2−^—which serve as alternative EAs—are commonly included in fertilizers to supply secondary or micronutrients to arable soils (Epstein and Bloom 2005; Hafeez et al. 2021). Thus, prior studies have demonstrated that these alternative EAs can effectively reduce methane (CH_4_) emissions in rice paddies by competitively suppressing methanogens and generating inhibitory intermediates (Bethke et al. 2011; Klüber and Conrad 1998; Sahrawat 2008; Yin et al. 2024). However, because NO_3_^−^ respiration yields the highest energy and is generally prioritized by microorganisms (Strohm et al. 2007), it has limited the exploration of alternative EAs for mitigating N_2_O emissions in upland arable soils. Contrary to traditional expectations, several studies have provided evidence for the simultaneous reduction of multiple terminal EAs, such as SO_4_^2−^ and Fe^3+^, under varying soil redox conditions (Jakobsen and Postma 1999; Wunder et al. 2021), and even concurrent Fe^3+^ reduction and methanogenesis (Schreiber et al. 2004). Field‐based observations further support these findings, demonstrating the concurrent use of multiple terminal EAs in natural soil systems (Canfield et al. 1993; Jørgensen et al. 2019; Vandieken et al. 2006).

Climate change, characterized by increased flooding and elevated irrigation demands due to intensified drought and rising temperatures, is expected to enhance conditions favorable for anaerobic microsites in upland soils. These changes may alter soil redox dynamics and promote the concurrent reductions of multiple terminal EAs (Freeman 2020; Xin et al. 2016). Under such fluctuating redox conditions, microorganisms may simultaneously activate terminal oxidase and N‐reductase pathways (Bourceau et al. 2023). Nevertheless, the interactions between alternative EA reduction and NO_3_^−^/NO_2_^−^ respiration pathways in upland arable soils remain poorly understood and warrant further investigation. Hence, in the heterogeneous environment of upland arable soils, we hypothesize that alternative EAs can function concurrently with NO_3_^−^ under transient anaerobic conditions. This simultaneous utilization of multiple EAs may alter the dynamics of EA competition and microbial prioritization, potentially reducing microbial N_2_O emissions.

To test this hypothesis, this study aimed to evaluate the effectiveness of alternative EAs in mitigating N_2_O emissions and to elucidate the underlying microbial and biogeochemical mechanisms. To assess the role of metal redox states (Fe^3+^, Mn^4+^, and SO_4_^2−^), we compared the effects of metal sulfates (MSs) with those of the corresponding zero‐valent metals (ZVMs). We conducted time‐batch microcosm experiments, quantified the expression of functional genes, characterized microbial communities, and measured concentrations of applied alternative EAs, along with isotope mapping. Additionally, 2‐year field experiments were conducted to monitor N_2_O fluxes and crop yield, thereby evaluating the practical feasibility of selected alternative EAs. Finally, to broadly assess the potential of viable EA, we performed a global‐scale meta‐analysis based on a systematic literature review.

## 2 Materials and Methods

### 2.1 Soil Incubation Experiment

#### 2.1.1 Soil Incubation Preparation

Soil samples were collected from an upland arable field at the experimental farm of Pusan National University in Miryang, Korea (35°30′08″ N 128°43′15″ E). The soil is classified as part of the *Bongsan* series (Fine loamy, mixed, mesic Typic Hapludults family), with its specific physical and chemical properties detailed in Table S1. The sampling site has been cultivated with maize for the past 6 years, including this study. Soil was collected from the top 15 cm, air‐dried at room temperature, sieved through a 2‐mm mesh to remove debris and stones, and homogenized. Subsequently, 400 g of the prepared soil was placed into each 860 mL glass microcosm jar.

To evaluate the role of metal redox states in N_2_O mitigation, we selected MSs (FeSO_4_, MnSO_4_, and ZnSO_4_) as candidate EAs and their corresponding ZVMs (Fe^0^, Mn^0^, and Zn^0^), alongside a no‐treatment control. The inclusion of ZVMs enabled us to isolate the effects of the metal components from those of SO_4_^2−^ and to examine their potential as alternative EAs. Metal sulfates and ZVM were applied at 0.01% (*w*/*w*), and urea [(NH_2_)_2_CO] was applied at 0.02% (*w*/*w*) on a dry‐weight basis to each microcosm jar. Soil moisture was adjusted and maintained at 65% WFPS, and jars were incubated in darkness at 25°C (±1.5) for 21 days. Prior to treatment, soils underwent a 10‐day pre‐incubation for microbial and physicochemical stabilization. For each incubation period (7, 14, and 21 days), 21 jars (3 replicates per treatment) were prepared, resulting in 63 microcosms. Jars remained open except during gas sampling.

The N application rate was established based on the recommended fertilization rate for maize cultivation (186 kg N ha^−1^) in the study region (National Institute of Agricultural Sciences 2022). This rate corresponds to approximately 0.015% N on a dry‐weight basis, assuming a bulk density of 1.20 g cm^−3^ and a 10 cm plow layer. To better distinguish treatment effects, we applied a slightly elevated N rate of 0.02%, while maintaining field‐relevant conditions. Unlike well‐defined macronutrients, guidelines for secondary and micronutrient application are less established. Previous studies suggest safe application ranges for secondary nutrients, including SO_4_^2−^ (50–2500 mg kg^−1^) and micronutrients, including iron, manganese, and zinc (20–600 mg kg^−1^) (Becker and Asch 2005; Dhaliwal et al. 2019; Epstein and Bloom 2005). Based on these recommendations, micronutrients were applied at 100 mg kg^−1^ (0.01%, *w*/*w*). Thus, our incubation experiment was conducted with a well‐defined application rate of 0.02% N and 0.01% selected EAs materials, ensuring a thorough and precise observation of their effects.

#### 2.1.2 Gas Sampling and N_2_O Measurements

Each jar was sealed with airtight rubber stoppers equipped with gas sampling ports. During incubation, gas samples (15 mL) were collected from jar headspaces at 0, 10, and 20 min daily. Nitrous oxide concentration was measured using a gas chromatograph‐mass spectrometer (GC–MS, QP2020, Shimadzu) equipped with an HP‐PLOT Q column, using helium as carrier gas (flow rate = 4.25 mL min^−1^). Calibration curves generated using certified N_2_O standards were used to determine sample concentrations. N_2_O flux (ng N_2_O g^−1^ h^−1^) was calculated as follows:

(1) $$N_{2}O\;\text{flux}\;\left(\mathrm{ng}\;N_{2}O\;g^{-1}\;h^{-1}\right)=\left(\Delta g/\Delta t\right)\times d\times \left(273/T\right)\times \left(V/W\right)$$

where, Δ*g*/Δ*t* represents the rate of change in N_2_O concentration inside the jar (g cm^−3^), *d* is the gas density (g m^−3^) at standard conditions (273 K and 0.101 Mpa), *T* is incubation temperature (K), *V* is the jar headspace volume (cm^3^), and *W* is the soil weight (400 g). Cumulative N_2_O emissions were calculated as follows:

(2) $$\text{Cumulative}\;N_{2}O\;\text{emissions}\;\left(\mathrm{\mu g}\;g^{-1}\;{\mathrm{day}}^{-1}\right)=\sum_{i}^{n}\left(R_{i}\times D_{i}\right)$$

Cumulative N_2_O emissions for the experimental period were calculated by multiplying the mean value of N_2_O emissions (N_2_O μg g^−1^ day^−1^) for each period (*R*_*i*_) by its duration (*D*_*i*_), then adding these values to the previous cumulative total.

#### 2.1.3 Nucleic Acid Extraction and Quantitative PCR

DNA and RNA were co‐extracted from time‐batch incubated soils, following the method described by Griffiths et al. (2000). To eliminate DNA, the extracted RNA was treated with TURBO DNase (Invitrogen, USA). The absence of DNA was confirmed through PCR (Bio‐Rad, Germany), which targeted the bacterial 16S rRNA gene. The purified RNA was then reverse transcribed to cDNA using LunaScript RT SuperMix (New England BioLabs GmbH, Germany), which includes random hexamer and poly‐dT primers to ensure coverage across the length of the RNA targets and an RNase inhibitor to prevent RNA template degradation. The quantity and quality of DNA and cDNA were measured using a NanoDrop (Allsheng, China) and stored at −20°C.

The abundance of various functional genes in the soil samples was assessed using qPCR on both DNA and cDNA. The targets included bacterial 16S rRNA; *amoA* and *hao* genes for nitrifiers; *nirK*, *nirS*, *norB*, and *nosZ* genes for denitrifiers; and the *dsrA* gene for sulfate‐reducing bacteria (SRB), serving as a proxy for transcript abundance in the soil. For each soil sample (*n* = 3), nested quantitative PCR (qPCR) was conducted using a CFX Connect Real‐Time PCR System (Bio‐Rad, Germany), resulting in six replicates per treatment and time point. Each qPCR assay (total volume = 20 μL) comprised 10 μL of 2 × AMPIGENE qPCR Green Mix Lo‐ROX (Enzo Life Sciences, USA), 2.0 μL each of forward and reverse primers (10 μM), 6 μL of DNase‐ and RNase‐free water (Thermo Fisher Scientific, Brunswick, Germany), and 2 μL of diluted template DNA or cDNA. The primers and qPCR conditions for targeting functional genes, along with amplification efficiencies (ranging from 89.4 to 111.0%), are provided in Table S2. Amplicon specificity was confirmed through melt curve analysis and 1.2% agarose gel electrophoresis. For absolute quantification of bacterial 16S rRNA and functional genes, plasmids containing the respective target gene sequences were constructed via PCR amplification and TA cloning using each primer set. Standard curves were generated from 10‐fold serial dilutions, and copy numbers were calculated as follows (Whelan et al. 2003).

#### 2.1.4 Soil Bacterial Sequencing and Bioinformatics Analysis

PCR amplification was conducted to construct sequencing libraries targeting the V3 to V4 regions of the 16S rRNA gene. The target region was amplified using the fusion primers 341F (5′‐AATGATACGGCGACCACCGAGATCTACAC‐XXXXXXXX‐TCGTCGGCAGCGTC‐AGATGTGTATAAGAGACAG‐CCTACGGGNGGCWGCAG‐3′) and 805R (5′‐CAAGCAGAAGACGGCATACGAGAT‐XXXXXXXX‐GTCTCGTGGGCTCGG‐AGATGTGTATAAGAGACAG‐GACTACHVGGGTATCTAATCC‐3′). The order of primer components is P5 (P7) graft binding, i5 (i7) index, Nextera consensus, adaptor, and target region (underlined sequences). The PCR conditions were as follows: initial denaturation at 95°C for 3 min, 25 cycles at 95°C for 30 s, 55°C for 30 s, and 72°C for 30 s, with a final elongation at 72°C for 5 min. The PCR products were confirmed using 1% agarose gel electrophoresis and visualized using a Gel Doc system (Bio‐Rad, USA). Subsequently, they were cleaned using CleanPCR (CleanNA, Waddinxveen, Netherlands), pooled in equal concentrations, and assessed for quality and size on a Bioanalyzer 2100 (Agilent, USA) using a DNA 7500 chip. Paired‐end sequencing (2 *×* 250 bp) was conducted by CJ Bioscience Inc. (CJ, Korea) on the Illumina MiSeq (Illumina, USA), following the manufacturer's protocols. The raw sequencing data were processed using Quantitative Insights into Microbial Ecology 2 (QIIME2, Version 2022.08) (Bolyen et al. 2019). Primer sequences were trimmed using the FASTX Toolkit (Version 0.0.14) (Liu et al. 2019), and demultiplexed sequences were merged using FLASH2 (Magoč and Salzberg 2011). The DADA2 plugin was used to denoise low‐quality reads (Q score < 25) and remove chimeric sequences. Overall, 545,414 reads were obtained from 16S rRNA gene amplicon sequencing, averaging 60,602 ± 6743 reads per soil sample (Table S3). After filtering low‐quality and chimeric reads, 372,414 reads were retained, averaging 41,379 ± 5,000 reads per soil sample. Good's coverage was above 99.5% in the soil samples (data not shown). Amplicon sequence variants (ASVs) were classified taxonomically using the SILVA 16S rRNA gene database (Version SSU138.1) (Quast et al. 2012), excluding unassigned ASVs, mitochondria, and chloroplast sequences. Alpha diversity metrics were calculated from rarefied tables with 31,118 ASVs per sample. Principal coordinate analysis (PCoA) was conducted based on unweighted and weighted UniFrac distances to assess dissimilarities in microbial communities. Shared and unique microbial taxa were visualized using the Venn diagram package in R (Version 4.2.1). The 16S rRNA gene sequencing data are available in the NCBI Sequence Read Archive (SRA) under BioProject No. PRJNA1107391 and SRA accession number SRX24441768‐76.

#### 2.1.5 Edaphic Characteristics

Extractable inorganic N ions (NH_4_^+^ and NO_3_^−^) and SO_4_^2−^ concentrations were quantified at incubation intervals (7, 14, and 21 days). For each time point, 5 g of wet soil was collected from each treatment, with a total of six replicates per treatment, including two technical replicates. Soil samples were extracted using 30 mL of 1 M KCl, a standard method for extracting plant‐ and microbial‐available forms of NO_3_^*−*^ and SO_4_^2−^, and then filtered through a 0.45 μm filter (Bloem et al. 2002; Kodithuwakku et al. 2024). Concentrations for NH_4_^+^ and NO_3_^*−*^ were analyzed using an autoanalyzer (Seal Analytical, Germany); SO_4_^2−^ were determined by ion chromatograph (Model ICS‐2000, Dionex Corporation, USA), and Fe^2+^ and Mn^2+^ were quantified via inductively coupled plasma mass spectrometer (Thermo Scientific iCAP Q, Germany). Consumption of extractable NO_3_^*−*^ and SO_4_^2−^ was calculated as the concentration difference between incubation for 7 days and 21 days.

#### 2.1.6 Isotope Mapping

To measure stable N_2_O isotopes (*δ* ^15^N^bulk^, *δ* ^18^O, and site preference [SP]), gas samples were collected from the headspace of the incubation jars (approximately 550 mL volume) on day 21. For each treatment, 15 mL of gas was extracted from each of the three replicates and pooled into 30 mL glass flasks (Pyrex, with stopcock). The pooled gas, after the 20‐min closure, was transferred using a vacuum line maintained with a turbo pump (~0.1 mTorr). Isotopic data were later corrected for ambient mixing effects based on mass balance equations. The isotopomer ratios of N_2_O were analyzed using a continuous‐flow isotope ratio monitoring mass spectrometer system (MAT252, Thermo Scientific, Burladingen, Germany) at the Tokyo Institute of Technology, as detailed by Toyoda et al. (2021). The measurement precision was ~0.2‰ for *δ* ^15^N^bulk^, ~0.3‰ for *δ* ^18^O, and ~0.5‰ for *δ* ^*15*^N^*α*^ and *δ* ^*15*^N^*β*^. The equations for expressing the isotopomer ratios are described below (Toyoda et al. 2008).
(3)

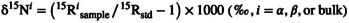



(4)

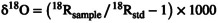



(5)

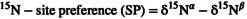




The N_2_O isotopic composition measured from the mixture of soil‐emitted air (δ_obs_) and its concentration (C_obs_) was corrected for atmospheric background mixing using ambient air data (δ_ambient_ and C_ambient_), which were estimated based on the 2009 average isotopomer ratios of N_2_O in the Northern Hemisphere, as reported in Toyoda et al. (2013). The correction was performed using the following mass balance equation (Toyoda et al. 2024):

$$\delta_{\mathrm{obs}}\times C_{\mathrm{obs}}=\delta_{\text{ambient}}\times C_{\text{ambient}}+\delta_{\text{soil}}\times C_{\text{soil}}$$

From this equation, δ_soil_ was derived to isolate the isotopic signature of N_2_O emitted solely from the soil. As a result of this correction, δ^15^N^bulk^ decreased by approximately ~3‰; δ^18^O values decreased by ~0.5‰, and SP decreased by ~1‰ on average, depending on the treatment.

### 2.2 Field‐Based Manipulation Experiment

#### 2.2.1 Field Experiment Design

This experiment evaluated the field‐scale feasibility of applying MSs to mitigate N_2_O emissions. The experiment was established in the same field, where soils for the incubation experiment were collected (Table S1) in a randomized complete block design with four replicates per treatment. Each plot measured 5 m × 5 m. Maize (*Zea mays L*.) was transplanted on July 25, 2018, and May 11, 2019, with a planting density of approximately 67,000 plants per hectare, based on an in‐row spacing of 25 cm and an inter‐row spacing of 60 cm. An annual fertilization regimen of 186–35–74 kg ha^−1^ (N‐P_2_O_5_‐K_2_O) was followed according to national recommendations (National Institute of Agricultural Sciences 2022). Fertilizer applications were split into two doses: half applied as basal fertilizer 10 days after transplanting and the other half at the seven‐ or eight‐leaf stage. Urea (46% N), potassium chloride (50% K_2_O), and superphosphate (12% P_2_O_5_) were used as fertilizer sources. Maize was harvested on October 4, 2018, and July 23, 2019.

Climate parameters, including air temperature, precipitation, irrigation events, and WFPS, were monitored throughout the 2‐year cultivation period (Figure S1). Weather data were obtained from a weather station located 1 km from the study site (Korea Meteorological Administration 2025). Supplemental irrigation was applied when rainfall was insufficient for maize establishment and growth. Daily volumetric soil moisture (*θ*, m^3^ m^−3^) at a 5 cm depth was measured using a soil moisture sensor (WT1000B, RF sensor, Korea) installed in each plot. Daily WFPS (%) was calculated as follows:

(6) $$\text{WFPS}=\left(\theta /\text{soil porosity}\right)\times 100$$

Soil porosity (m^3^ m^−3^) was estimated from monthly bulk density measurements, assuming a particle density of 2.65 g cm^−3^.

The MSs were applied at 0 and 20 kg ha^−1^ rates, with the applications evenly broadcast near maize plants on May 16, 2018, and May 5, 2019. Although standard application methods for secondary and micronutrients are not well defined, the American Plant Food Control Officials (AAPFCO) recommends a minimum micronutrient rate of 5–10 kg ha^−1^, including Fe, Mn, and Zn, with higher rates often necessary for agronomic effectiveness (AAPFCO 2012). Similarly, sulfate application rates of 20–40 kg ha^−1^ or greater are advised where deficiencies exist (Amissah et al. 2024). No phytotoxic effects have been reported at these levels (Gupta and Gupta 1998), indicating that the applied rate was both effective and environmentally safe. Thus, a 20 kg ha^−1^ rate was selected as a balanced, agronomically appropriate, and environmentally sustainable application rate for this experiment.

#### 2.2.2 N_2_O Sampling, Measurements, and Calculation

N_2_O emissions were measured using the static closed‐chamber method, which was modified and validated by our research group and officially adopted into national guidelines (Choi et al. 2023). PVC anchors equipped with removable lids were permanently installed in the field. Each chamber lid was placed over the chamber during gas sampling events. Gas samples (30 mL each) were collected from the chamber headspace at 0, 20, and 40 min after lid placement, typically between 10:00 a.m. and 12:00 p.m. Sampling was conducted once per week throughout the 2‐year period. To better capture N_2_O flux dynamics during periods of high emission potential, such as after fertilizer and treatment applications, irrigation, rainfall, and harvest, the sampling frequency was increased to twice per week. Samples were immediately transferred into 12 mL evacuated glass vials (Vial‐evacuated 838 W, Labco, UK). N_2_O concentrations were analyzed using the same procedure as described in Section 2.1.2. According to Equations (1) and (2), N_2_O flux and cumulative N_2_O emissions were calculated and adjusted using the closed‐chamber method for field‐based experiments. The equations were modified to reflect the specific dimensions of the field chambers, including a headspace volume of 1500 cm^3^ and a surface of 0.032 m^2^, based on PVC lids with a diameter of 20.2 cm and a height of 17 cm. The area convection coefficient was set at 10.2 cm^−2^. To determine yield‐scaled N_2_O emissions (YSNE), the cumulative N_2_O emission was divided by the dried maize ear yield (MEY) harvested from each plot as follows:

(7) $$\text{YSNE}\;\left(\mathrm{kg}\;N_{2}O\;{\mathrm{Mg}}^{-1}\;\text{yield}\right)=\frac{\text{Cumulative}\;N_{2}O\;\text{emission}\;\left(\mathrm{kg}\;N_{2}O\;{\mathrm{ha}}^{-1}\;{\mathrm{yr}}^{-1}\right)}{\text{Dried maize}\;\mathrm{ear}\;\text{yield}\;\left(\mathrm{Mg}\;{\mathrm{ha}}^{-1}\;{\mathrm{yr}}^{-1}\right)}$$

### 2.3 Literature Review and Data Collection

We undertook a thorough systematic literature review, adhering strictly to PRISMA guidelines to maintain high methodological rigor, transparency, and reproducibility standards. By utilizing the keywords ‘(Sulfate) AND (Nitrous Oxide) AND (Soils)’, we explored relevant publications in the Web of Science database. A total of 327 records were initially identified (search conducted on March 1, 2025). Our selection criteria were rigorous: (1) exclusion of reviews, meta‐analyses, or model‐based studies; (2) inclusion of only field or controlled soil incubation studies that reported cumulative N_2_O emissions; and (3) exclusion of experiments involving non‐upland or non‐agricultural soils, such as rice paddies and wetlands. We also excluded SO_4_^2−^ typically applied with N fertilizers, such as ammonium sulfate [(NH_4_)_2_SO_4_], since their co‐application with N can increase N_2_O emissions, making it challenging to isolate sulfate's role as an alternative EA. Exceptionally, we included studies using (NH_4_)_2_SO_4_ as a SO_4_^2−^ source only when they offered a control treatment with an equivalent N amount without SO_4_^2−^. This approach enabled us to assess the effects of SO_4_^2−^ on N_2_O emissions distinctly. We gathered 74 data points from 19 published studies and our field experiment. Key information extracted from the selected studies included the type of SO_4_^2−^ material, the SO_4_^2−^ application rate, the crop type, and the cumulative N_2_O emissions for both treatment and control groups. Figure data were digitized using WebPlotDigitizer (Version 4.6, Pacifica, USA). We compared the response ratio of N_2_O emissions from sulfate‐treated soils and control soils without SO_4_^2−^ application, under otherwise comparable conditions. In cases where multiple controls were available (e.g., unfertilized vs. conventionally fertilized), we selected the control group that received the same nitrogen fertilization as the sulfate‐treated group.

### 2.4 Statistical Analysis

Statistical analyses were conducted using R Studio (Version 3.4.4, R Core Team, Austria) with the “Agricolae” package. Treatment effects on N_2_O flux, cumulative N_2_O emissions, MEY, YSNE, exchangeable NH_4_^+^, NO_3_^−^, and SO_4_^2−^ concentrations, and the copy numbers of functional genes (*amoA*, *hao*, *nirK*, *nirS*, *norB*, *nosZ*, and *dsrA*) were evaluated using one‐way ANOVA. Pairwise treatment comparisons were performed using the least significant difference (LSD) test, applied only when the overall *F*‐test indicated significant differences (*p* < 0.05). In the global‐scale meta‐analysis based on a systematic literature review, the 95% confidence interval (CI) of the response ratio for cumulative N_2_O emissions was calculated using a one‐sample *t*‐test, which assessed whether the mean response ratio significantly differed from zero.

Data from 16S rRNA sequencing did not conform to the normal distribution, even after several transformations, including log, square root, and arcsine. Therefore, nonparametric analyses were employed. For alpha diversity measurements, the Kruskal–Wallis rank sum test was used to identify significant differences among treatments (control, ZVM, and MS) using the kruskal.test function in the stats package in R. If a significant difference was observed in the Kruskal–Wallis rank sum test, Dunn's post hoc test with the Benjamini–Hochberg adjustment was conducted using the dunnTest function in the FSA package (Ogle et al. 2023). Permutational multivariate analysis of variance (PERMANOVA) and permutational multivariate analysis of dispersion (PERMDISP) with 9999 random permutations were conducted in QIIME2 to compare differences in soil microbiota among treatments and within‐group variances. Analysis of Compositions of Microbiomes with Bias Correction 2 (ANCOM‐bc2) was used to identify differentially predominant phyla and genera (Lin and Peddada 2020). Spearman correlation coefficients between soil properties and differentially abundant microbial taxa identified from ANCOM‐bc2 were computed using the microbiome package (Lahti et al. 2014) and visualized in R Studio. Spearman correlation analysis was conducted to identify potential links between differentially abundant genera and soil properties using the R package microbiome and visualized using the R package “ggplot2”. Statistical significance was defined as *p* < 0.05, while values between 0.05 and 0.10 were speculated as statistical trends.

## 3 Results

### 3.1 N_2_O Fluxes

**FIGURE 1:**
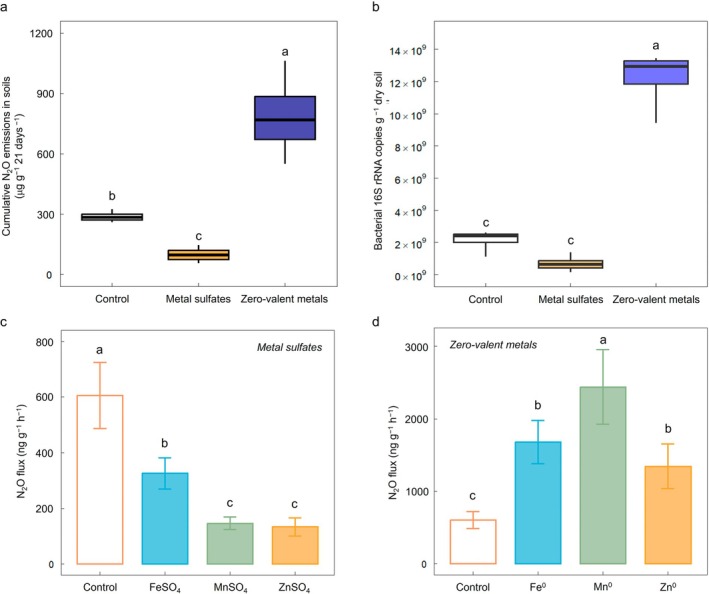
Comparative effect of metal sulfates and zero‐valent metals (ZVMs) in soil microcosm experiments. (a) The mean value of cumulative N_2_O emissions, (b) bacterial 16S rRNA gene abundance across treatments including control, metal sulfates, and ZVMs, (c) N_2_O flux comparison for metal sulfates (control, FeSO_4_, MnSO_4_, and ZnSO_4_), and (d) N_2_O flux for ZVMs (control, Fe, Mn, and Zn) after 21 days of incubation. Statistically significant differences among the treatment groups based on one‐way ANOVA by the least significant difference test (*p* < 0.05) are indicated by differing letters.

MS and ZVM treatments significantly influenced N_2_O emissions (Figure 1a). ZVM application notably increased cumulative N_2_O emissions, whereas MS application effectively minimized them. Specifically, MnSO_4_ and ZnSO_4_ treatments resulted in the most substantial decrease in N_2_O fluxes, closely followed by FeSO_4_. After 15 days of incubation, MS treatments achieved a 46%–78% decrease in N_2_O flux compared to the control (0.61 μg g^−1^ h^−1^, Figure 1c). Conversely, ZVM treatments significantly elevated N_2_O fluxes, ranging from 1.35 to 2.44 μg g^−1^ h^−1^ (Figure 1d). This pattern aligned with the cumulative N_2_O emissions observed over time in the microcosm experiment, which showed consistent trends across all treatments without significant temporal variation (Figure S2). This study sought to uncover the mechanisms responsible for reducing N_2_O emissions. To achieve this, we focused our further analyses—such as inorganic N concentrations, qPCR‐based functional gene abundances, and differential abundance testing—only on the MS treatments, which consistently displayed notable N_2_O mitigation effects. In stark contrast, the ZVM treatments caused a significant spike in N_2_O emissions, leading to their exclusion from subsequent analyses to identify effective mitigation pathways.

### 3.2 Isotope Mapping

**FIGURE 2:**
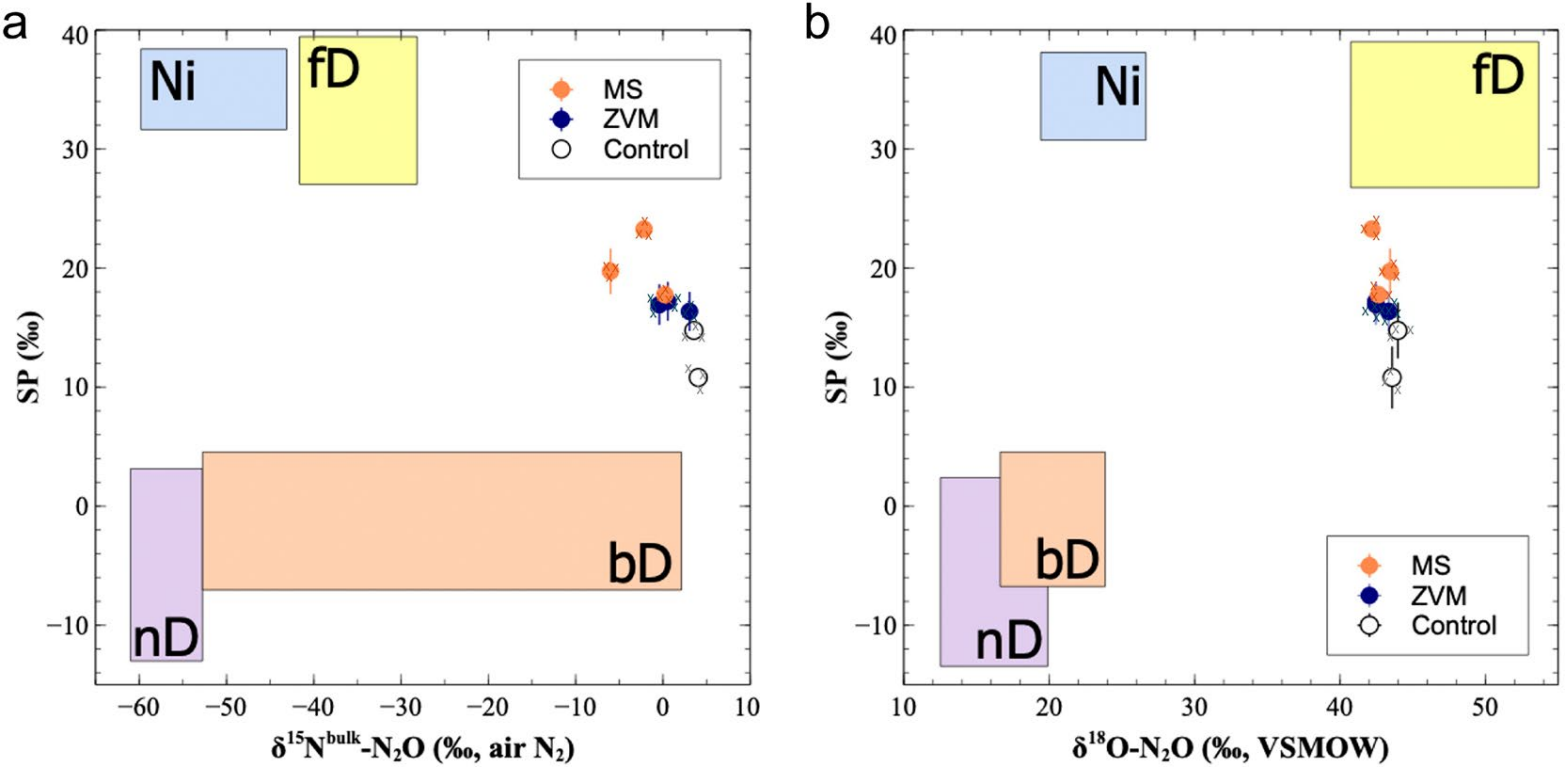
Dual isotope mapping of N_2_O to identify dominant microbial pathways of production across treatments. (a) Isotope map using site preference (SP) and δ^15^N^bulk^ and (b) isotope map using SP and δ^18^O. Each point represents the average isotopic value of three mixture combinations per treatment. Domains for nitrification (Ni), bacterial denitrification (bD), nitrifier denitrification (nD), and fungal denitrification (fD) are delineated based on end‐member values from Yu et al. (2020).

To investigate the pathways of N_2_O production in the studied soils, we analyzed the stable isotopic composition of N_2_O collected from soil air samples and employed dual isotope mapping, referencing established isotopic signatures from Yu et al. (2020) (Figure 2). The experiment consisted of three treatments: control, MS, and ZVM. Each isotopic data point represents the average value derived from three mixture combinations within each treatment group. The control treatment exhibited δ^15^N^bulk^, δ^18^O, and SP values of 3.8‰ ± 0.4‰, 43.8‰ ± 0.3‰, and 12.8‰ ± 2.8‰, respectively. In comparison, the MS treatment showed δ^15^N^bulk^, δ^18^O, and SP values of −2.7‰ ± 3.1‰, 42.8‰ ± 0.6‰, and 20.3‰ ± 2.8‰, respectively. The ZVM treatment exhibited δ^15^N^bulk^, δ^18^O, and SP values of 1.1‰ ± 1.8‰, 42.8‰ ± 0.5‰, and 16.8‰ ± 0.4‰, respectively. These isotopic patterns suggest that heterotrophic denitrification was more prominent in the control, consistent with its lower SP values overlapping the bacterial denitrification (bD) domain (Figure 2a,b). Both MS and ZVM treatments exhibited decreased δ^15^N^bulk^ relative to the control, but the extent of depletion was more pronounced in the MS. Additionally, the MS treatment displayed distinctly elevated SP values compared to both control and ZVM treatments, suggesting a shift towards nitrification or suppression of N_2_O reduction processes. In contrast, ZVM samples exhibited only minor differences from the control in SP and δ^15^N^bulk^, suggesting limited changes in microbial N_2_O production or reduction pathways. Across all treatments, δ^18^O values remained relatively stable, indicating that oxygen isotope composition was less sensitive to the treatments applied.

### 3.3 Ammonium, Nitrate, and Sulfate Concentrations

**FIGURE 3:**
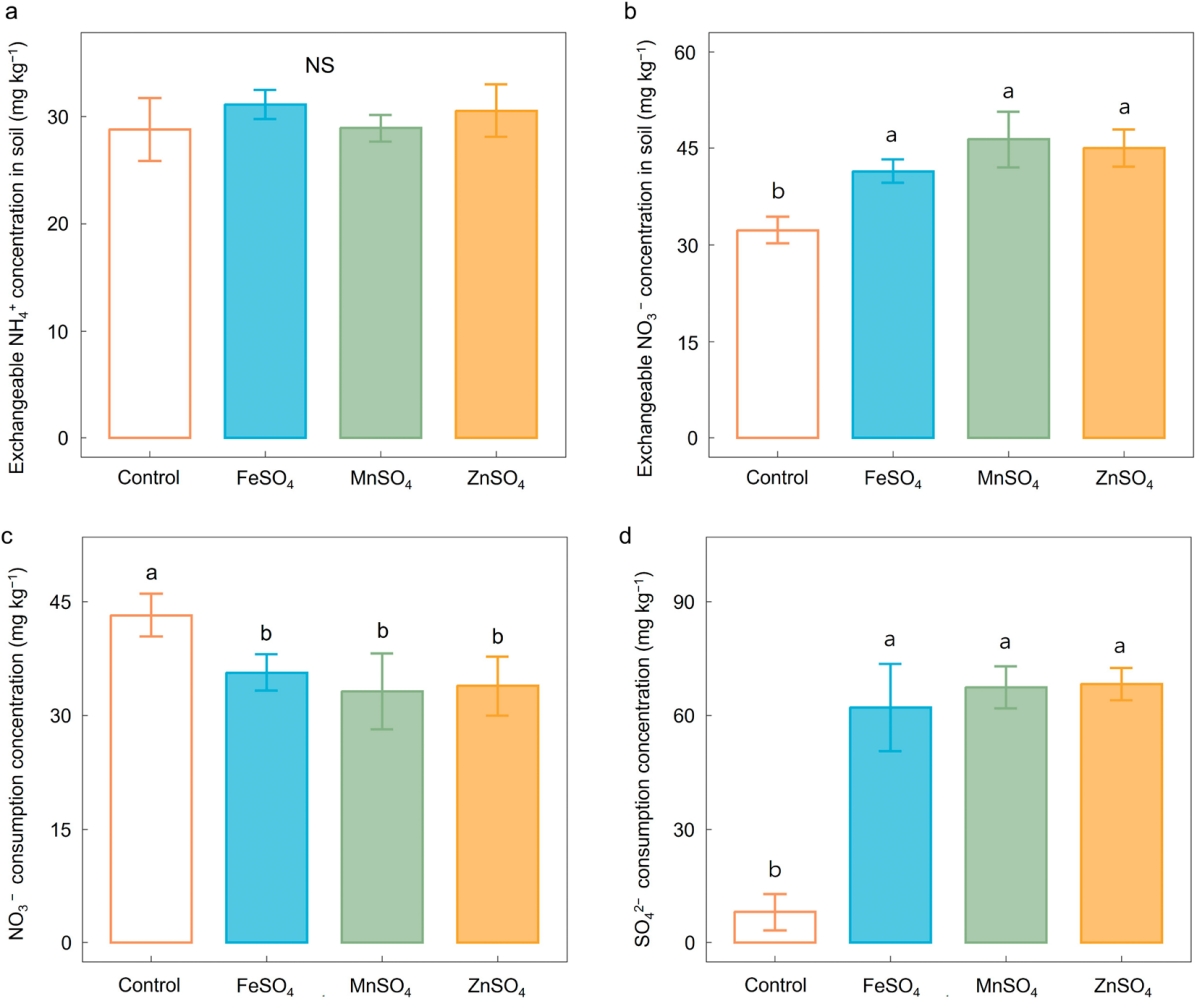
Changes in soil ion concentration affected by metal sulfates. Extractable (a) NH_4_^+^ and (b) NO_3_^−^ concentrations in soil with metal sulfates (FeSO_4_, MnSO_4_, ZnSO_4_, and control) after a 21‐day incubation period. Net consumption of (c) NO_3_^−^ and (d) SO_4_^2−^ in soils with metal sulfate treatments by determining the difference in each concentration between the 21‐ and 7‐day incubation periods. Statistically significant differences among the treatment groups based on one‐way ANOVA by the least significant difference test (*p* < 0.05) are indicated by differing letters.

Ammonium and NO_3_^−^, transformed from urea, are crucial substrates for N_2_O production via nitrification and denitrification in soil. After 21 days of incubation, exchangeable NH_4_^+^ concentrations did not differ significantly between the MS‐treated soils and the control (Figure 3a). In contrast, exchangeable NO_3_^−^ concentrations were significantly higher in soils treated with MSs compared to the control, reaching 41.5, 46.4, and 45.0 mg kg^−1^ for FeSO_4_, MnSO_4_, and ZnSO_4_, respectively, versus 32.3 mg kg^−1^ in control (*p* < 0.05, Figure 3b). Correspondingly, NO_3_^−^ consumption was significantly minimized in the MS‐treated soils, with consumption rates of 35.6, 32.5, and 33.8 mg kg^−1^ for FeSO_4_, MnSO_4_, and ZnSO_4_, respectively, compared to 43.1 mg kg^−1^ in control (*p* < 0.05, Figure 3c). Sulfate consumption was also significantly greater in MS‐treated soils (Figure 3d). The control exhibited the lowest SO_4_^2−^ consumption at 8.03 mg kg^−1^, whereas FeSO_4_, MnSO_4_, and ZnSO_4_ application showed substantially higher SO_4_^2−^ consumption levels of 63.5, 67.8, and 69.1 mg kg^−1^, respectively (*p* < 0.05). These results indicate that MS application effectively enhanced SO_4_^2−^ reduction. Conversely, decreases in exchangeable Fe and Mn concentrations in FeSO_4_ and MnSO_4_ applications were relatively minor (13.5 and 14.3 mg kg^−1^, respectively) and statistically insignificant (*p* = 0.32 and 0.41, respectively; data not shown).

### 3.4 Functional Gene Expressions

**FIGURE 4:**
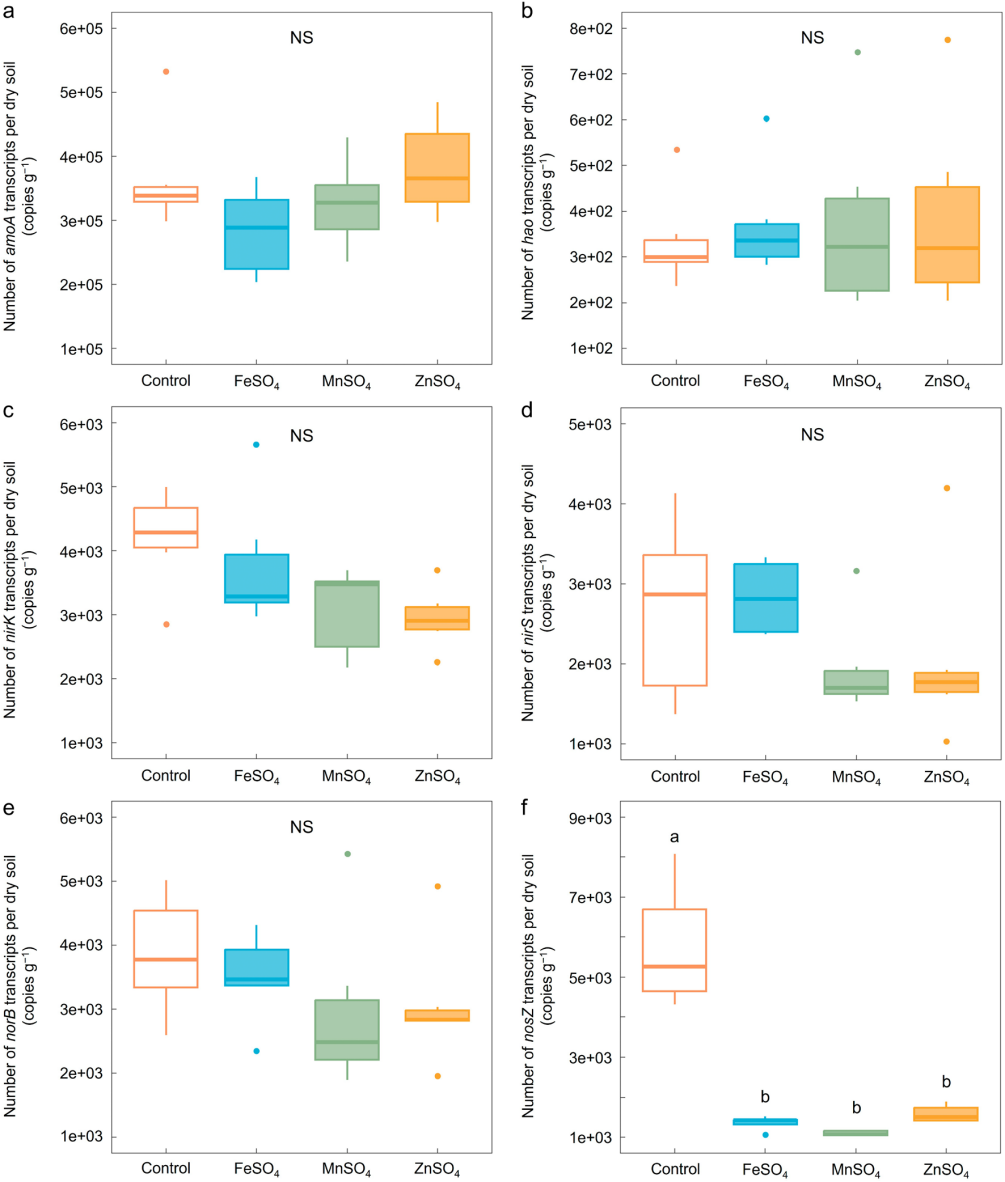
Impact of metal sulfates on functional gene expression in the nitrogen cycle. These figures illustrate the transcript abundance of (a) bacterial *amoA*, (b) *hao*, (c) *nirK*, (d) *nirS*, (e) *norB*, and (f) *nosZ* genes per gram of dry soil treated with metal sulfates after a 21‐day incubation period. Each box represents the mean copy numbers for treatments with control, FeSO_4_, MnSO_4_, and ZnSO_4_. The thick central line represents the median value, the boxed areas represent the interquartile range, and the spots show the maximum and minimum values (*N* = 6). Statistically significant differences among the treatment groups, determined by one‐way ANOVA with the least significant difference test (*p* < 0.05), are indicated by differing letters.

The absolute abundance of bacterial 16S rRNA genes across treatments reflected the observed trends in cumulative N_2_O emissions (Figure 1a,b), which aligns with previous studies that identified bacterial activity as a key determinant of N_2_O emissions (Butterbach‐Bahl et al. 2013; Decock and Six 2013; Philippot et al. 2011). Specifically, the ZVM treatments significantly increased bacterial 16S rRNA gene abundance compared to the control, whereas MS treatments exhibited no notable change (*p* < 0.01, Figure 1b). The abundance of *amoA*, *hao*, *nirK*, *nirS*, and *norB* mRNAs in MS treatments remained largely unchanged (not significant, Figure 4a–e). Nevertheless, a consistent decreasing trend in *nirK*, *nirS*, and *norB* gene expression was noted, indicating moderate inhibition of denitrification activity under MS treatments. Additionally, the abundance of *nosZ* mRNA under MS treatments (ranging between 1.07 × 10^4^ and 1.58 × 10^4^ copies per gram of dry soil) was significantly lower compared to the control (5.75 × 10^4^) (*p* < 0.05, Figure 4f), suggesting a potent inhibition of complete denitrification.

### 3.5 Microbial Communities

**FIGURE 5:**
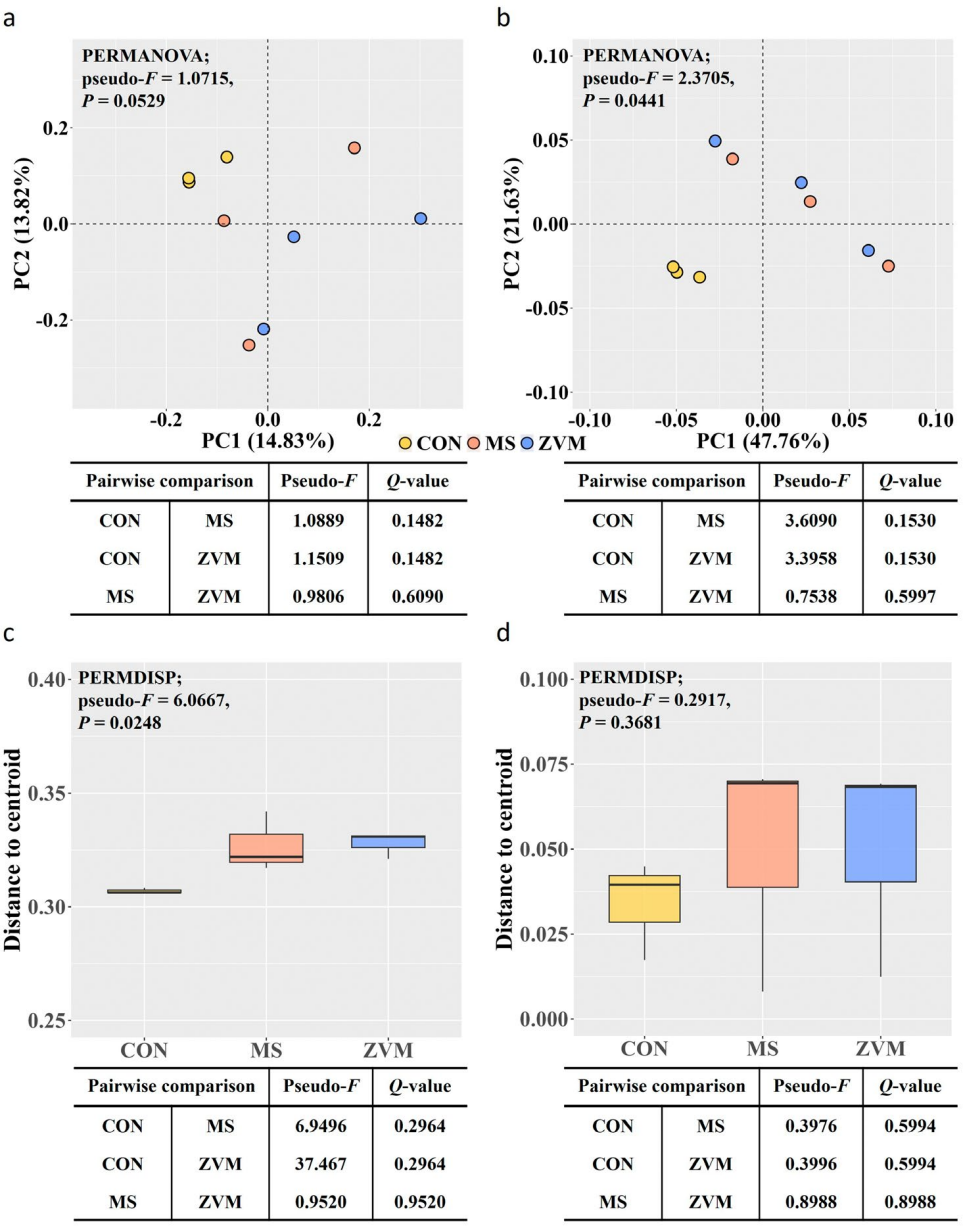
Comparison of microbial community structures influenced by treatment groups. Treatment abbreviations: CON (control), MS (metal sulfate), and ZVM (zero‐valent metal). Principal coordinates analysis based on (a) unweighted and (b) weighted UniFrac distances of soil microbiota. Permutational multivariate analysis of variance (PERMANOVA) was conducted to compare differences in soil microbiota among treatments. Boxplot of homogeneity of group dispersions based on (c) unweighted and (d) weighted UniFrac distances. Permutational multivariate analysis of dispersion (PERMDISP) was used to compare within‐group variances.

**FIGURE 6:**
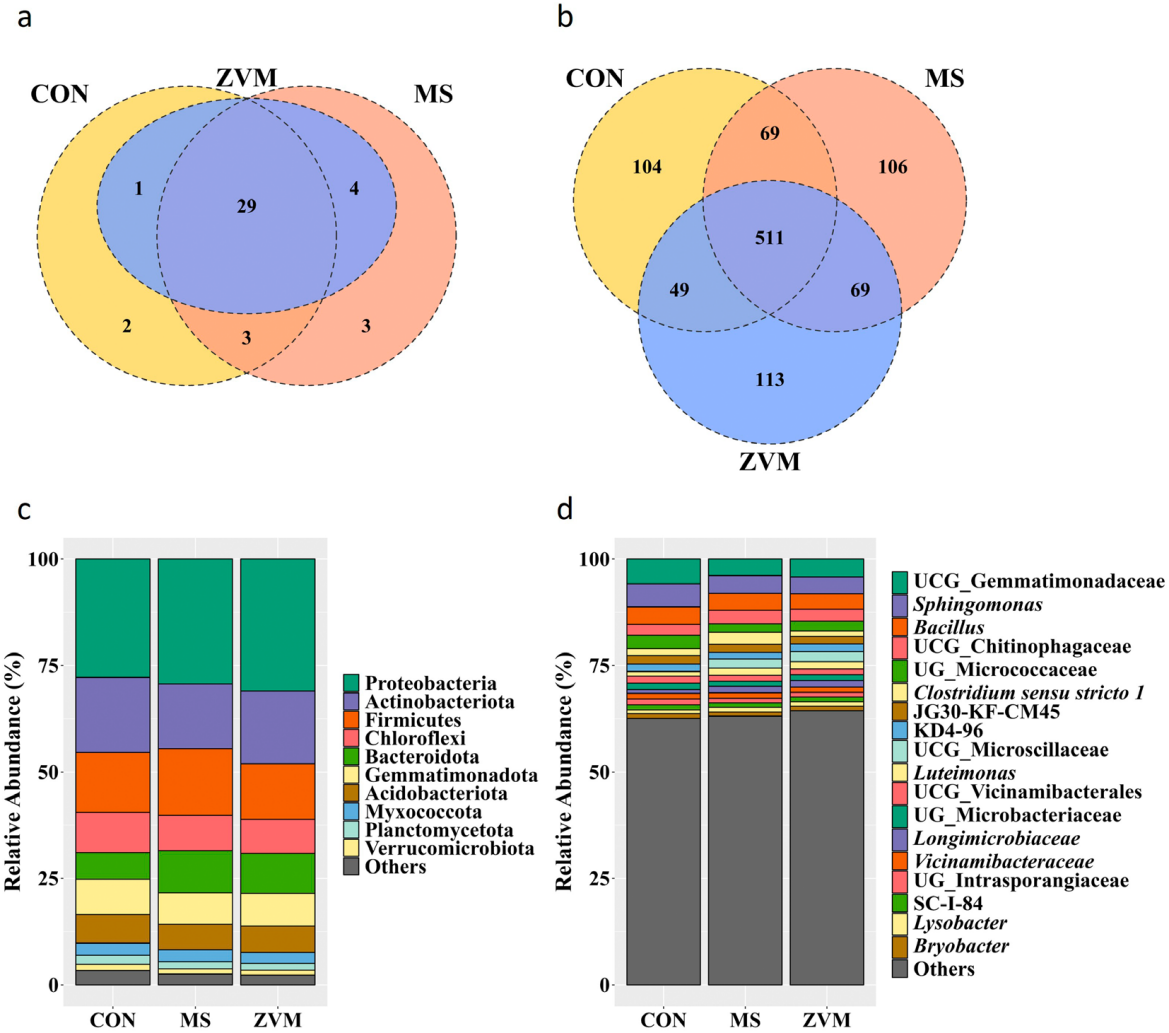
Distribution of core and distinct microbial taxa across treatment groups at phylum and genus levels. Treatment abbreviations: CON (control), MS (metal sulfate), and ZVM (zero‐valent metal). Venn diagram depicting shared and unique microbiota at (a) phylum and (b) genus levels. Predominant soil microbiota at the (c) phylum and (d) genus levels. Only taxa showing ≥ 30% prevalence and 1% relative abundance were visualized.

Across all alpha diversity measurements, the alpha rarefaction curves plateaued as sequencing depth increased, indicating sufficient sequencing coverage for all samples (Figure S3). No significant differences were observed in all alpha diversity measurements (observed ASVs, Chao1, Evenness, Faith's phylogenetic diversity, Shannon index, and Simpson index) among treatment groups (Figure 5, *p* > 0.10). Although overall differences in microbial community structures were detected among treatment groups (PERMANOVA: *p* = 0.053 for unweighted UniFrac; *p* = 0.044 for weighted UniFrac) and within‐group dispersion showed significance in unweighted distances (PERMDISP: *p* = 0.025), post hoc pairwise comparisons did not reveal statistically significant differences between any two treatment groups (*Q* > 0.05) (Figure S4). Venn diagram analysis revealed that 69.1% of phyla and 50.1% of genera were shared among the treatment groups (Figure 6a,b). At the phylum level, all microbial taxa detected in the ZVM group overlapped with those found in the control and/or MS groups (Figure 6a), whereas the ZVM group exhibited the highest number of unique genera (Figure 6b). The microbial taxa detected in at least 30% of occurrences and with a relative abundance of at least 0.1% among the soil samples were defined as core microbial taxa. The main phyla were primarily *Proteobacteria*, *Actinobacteriota*, and *Firmicutes*, accounting for an average of 60% of total sequences (Figure 6c). At the genus level, *UCG_Gemmatimonadaceae*, Sphingomonas, and *Bacillus* were the most abundant genera in amplicon libraries, constituting 11.8% of the total soil microbiota sequences across the samples (Figure 6d). Additionally, 18 genera at an average of 1% in relative abundance were assigned to seven different phyla: *Gemmatimonadota*, *Proteobacteria*, *Firmicutes*, *Bacteroidota*, *Actinobacteriota*, *Chloroflexi*, and *Acidobacteriota*, which indicates substantial variations at the genus level despite the similarities observed at the phylum level.

**FIGURE 7:**
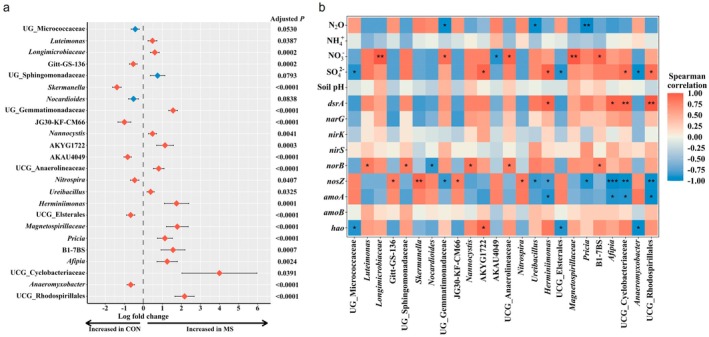
Differences in major soil microbiota by metal sulfate addition. Treatment abbreviations: CON (control) and MS (metal sulfate). (a) Differentially abundant genera identified from the Analysis of Compositions of Microbiomes with Bias Correction 2 (ANCOM‐bc2). Only genera detected with at least ≥ 30% prevalence and 0.1% relative abundance across the treatments were evaluated. Data are represented by log fold change ±95% confidence interval. Red or blue symbols represent significant differences or statistical tendencies, respectively. (b) Spearman correlation analysis between differentially abundant genera, functional genes, and soil properties. Only those with significant and strong coefficients ($\left|\rho \right|$ > 0.7, *p* < 0.05) were visualized using asterisks: *p ≤ 0.05; **p ≤ 0.01, ***p ≤ 0.001. UG_, unclassified genus; UCG_, uncultured genus.

Through ANCOM‐bc2 analysis, we identified 24 differentially abundant genera between the control and MS treatments (Figure 7a and Table S4). In the control group, 9 genera were enriched, including unclassified genus (*UG*) *Micrococcaeceae*, *Gitt‐GS‐136*, *Skermanella*, *Nocardioides*, *JG30‐KF‐CM66*, *AKAU4049*, *Nitrospira*, uncultured genus (*UCG*) *Elsterales*, and *Anaeromyxobacter*. Interestingly, 7 out of 15 differentially abundant genera in the MS group belonged to the phylum *Proteobacteria*, i.e., *Luteimonas*, *UG_Sphingomonadaceae*, *Afipia*, *B1‐7BS*, *Herminiimonas*, *Magnetospirillaceae*, and *UCG_Rhodospirillales*. To further understand the ecological implications of these shifts, Spearman correlation analysis was conducted between the differentially abundant genera and soil physicochemical properties and N‐cycle functional genes (Figure 7b). Dominant community members in MS‐treated soils, such as *Gemmatimonadaceae*, *Ureibacillus*, and *Pricia*, were negatively correlated with N_2_O. *Herminiimonas*, *Cyclobacteriaceae*, and *Rhodospirillales*, which exhibited increased relative abundance in MS treatments, also showed a negative correlation with *nosZ*.

### 3.6 N_2_O Fluxes and Crops Yield Under Field Conditions

In field‐based manipulation experiments, daily N_2_O fluxes with all MS treatments exhibited consistent patterns aligned with agricultural practices across 2 years (Figure S5). MS treatments significantly influenced cumulative N_2_O emissions (*p* < 0.05, Table 1). Average cumulative N_2_O emissions varied between the two experimental years, with 4.81 kg ha^−1^ in 2018 (Y1) and 3.76 kg ha^−1^ in 2019 (Y2). Over 2 years, the control exhibited the highest cumulative N_2_O emissions, followed by MnSO_4_, FeSO_4_, and ZnSO_4_ treatments. MS application consistently decreased N_2_O emissions relative to the control, achieving reductions ranging from 9.6% to 24.3%. Notably, decreases in N_2_O emissions associated with FeSO_4_ and ZnSO_4_ were statistically significant (*p* < 0.05), whereas MnSO_4_ did not achieve statistical significance. The 95% CIs for cumulative N_2_O emissions were 4.64–5.00 kg ha^−1^ for the control and 3.95–4.79, 3.85–4.31, and 3.39–4.30 kg ha^−1^ for MnSO_4_, FeSO_4_, and ZnSO_4_, respectively.

**TABLE 1:** Feasibility of metal sulfates as a soil amendment in field‐based manipulation experiments.

Metal sulfates (MSs)	Cumulative N_2_O emissions (kg ha^−1^)			Maize ear yield (Mg ha^−1^)			Yield‐scaled N_2_O emissions (kg Mg^−1^)		
	Y1	Y2	MS mean	Y1	Y2	MS mean	Y1	Y2	MS mean
Control	5.52^a^	4.12^a^	4.82^a^	6.81^b^	5.32^a^	6.07^a^	0.81^a^	0.77^a^	0.79^a^
Iron sulfate	4.55^b^	3.55^b^	4.05^b^	6.74^b^	5.53^a^	6.14^a^	0.67^b^	0.64^b^	0.66^b^
Manganese sulfate	4.99^b^	3.75^b^	4.37 ^ab^	7.28^a^	4.93^a^	6.11^a^	0.68^b^	0.75^ab^	0.72^b^
Zinc sulfate	4.18^b^	3.60^b^	3.89 ^b^	7.19^ab^	5.53^a^	6.36^a^	0.58^b^	0.65^b^	0.62^b^
Year mean	4.81^A^	3.76^B^		7.16^A^	5.33^B^		0.69^A^	0.71^A^	

*Note:* This table presents cumulative N_2_O emissions, maize ear yield, and yield‐scaled N_2_O emissions for metal sulfate treatments across two field experiment years, 2018 (Y1) and 2019 (Y2). The “MS mean” column represents the average values for each metal sulfate treatment over the 2 years, while the “Year mean” denotes the average across all treatments for each year. Statistical differences between treatments within a year are indicated by lowercase letters, and differences between the 2 years by uppercase letters, based on one‐way ANOVA by the least significant difference test (*p* < 0.05).

The mean MEY over the 2 years did not differ significantly between the control and MS treatments (Table 1). The average MEY was 7.16 Mg ha^−1^ in Y1 and 5.33 Mg ha^−1^ in Y2, ranging from 4.93 to 7.28 Mg ha^−1^. These values fall within the midrange of the MEYs reported by researchers in similar regions using the same NF rates and cropping systems in upland arable soils, which have an average of 5.82 ± 2.08 Mg ha^−1^ (Huang et al. 2017; Park et al. 2022). Over the 2 years, YSNE in MS treatments was significantly lower than in the control (*p* < 0.05, Table 1). While the control had an average YSNE of 0.79 kg Mg^−1^, MS treatments resulted in significantly lower YSNE values, ranging from 0.62 to 0.72 kg Mg^−1^, indicating a minimum of 7.6%–21.5%. This decrease in N_2_O emissions and a slight increase in MEY contributed to the significantly lower YSNE in the MS treatments than the control. Nitrogen uptake by crops (grain, stem, and leaf) was also measured over the 2‐year experimental period; however, no statistically significant differences were observed among the treatments (Table S5).

## 4 Discussion

### 4.1 Contrasting Effects of Electron Acceptors and Donors on N_2_O Emissions

We monitored N_2_O emissions in a time‐batch incubation experiment to assess how MS and ZVM applications influenced N_2_O fluxes in upland arable soils. Our findings revealed clear contrasts between treatments. Specifically, ZVM applications significantly increased N_2_O emissions (Figure 1a). The typical oxidation reactions of ZVM in soils can be summarized as follows:

(9) $${\mathrm{Fe}}^{0}\to {\mathrm{Fe}}^{2+}+{\text{2e}}^{-}\to {\mathrm{Fe}}^{3+}+e^{-}$$

(10) $${\mathrm{Mn}}^{0}\to {\mathrm{Mn}}^{2+}+{\text{2e}}^{-}\to {\mathrm{Mn}}^{4+}+{\text{2e}}^{-}$$

(11) $${\mathrm{Zn}}^{0}\to {\mathrm{Zn}}^{2+}+{\text{2e}}^{-}$$

All ZVMs acted as electron donors and stimulated N_2_O emissions according to the number of electrons released during their oxidation. Mn^0^ exhibited the highest N_2_O flux, followed by Fe^0^ and Zn^0^, which correlated with the number of electrons each metal can release during oxidation into the soil (Figure S1). These findings suggest that the electrochemical properties of these metals significantly influenced their effects on N_2_O emissions. Additionally, Fe, Mn, and Zn in soil may stimulate N_2_O production due to their role as cofactors in microbial enzymes associated with the N cycle. Iron plays a crucial role in electron transport chains essential for microbial transformations, including reducing NO_3_^−^ in denitrification processes (Xin et al. 2016). Manganese is essential for superoxide dismutase in oxidative stress response and Zn^2+^ for metalloproteases (Su et al. 2016). However, such metal ions rarely limit microbial activity in soils, and N‐cycle processes are not more demanding than other microbial processes. Thus, data suggest that ZVM stimulated N_2_O emissions due to the alleviation of the electron donor limitation of denitrification.

Applying MSs emerged as an effective strategy to reduce N_2_O emissions in upland arable soils, with ZnSO_4_ exhibiting the greatest mitigation effect (Figure 1c). Zinc sulfate was deliberate, considering that Zn^2+^ is not typically an EA and its molecular weight is comparable to Fe and Mn, to isolate and examine the effects of SO_4_^2−^ alone. We initially anticipated FeSO_4_ and MnSO_4_, with their synergistic combination of alternative EAs from cation and anion, to be more effective in reducing N_2_O emissions. This expectation is based on the ability of divalent metal ions (Fe^2+^ and Mn^2+^) to oxidize to Fe^3+^ and Mn^4+^ in upland arable soils, offering higher energy yields than SO_4_^2−^ under oxygen‐limited conditions (Bethke et al. 2011). However, our results indicate that SO_4_^2−^ was preferentially utilized, reducing N_2_O emissions more effectively than treatments including Fe or Mn.

The limited effectiveness of Fe and Mn as EAs in our study is likely attributed to their low bioavailability in upland arable soils. Even if Fe^3+^ and Mn^4+^ are present, these oxidized forms quickly become insoluble due to their strong adsorption to soil particles or precipitation as compounds such as ferric hydroxide, goethite, hematite, and manganese dioxide (Hem 1963; Lindsay 1979b; Schwertmann and Murad 1983). In contrast, sulfate‐based materials exhibit superior solubility and availability for microbial utilization. Sulfates are highly water‐soluble, allowing them to readily diffuse in soil solution and become accessible to microorganisms, unlike Fe and Mn oxides, which often remain in insoluble forms (Charlet et al. 1993). Another advantage of SO_4_^2−^ as an EA in upland arable soils is its regenerative nature. While SO_4_^2−^ reduction results in the formation of S^2−^, periodic aeration can re‐oxidize S^2−^ back to SO_4_^2−^ (Eimers et al. 2003), maintaining its availability in the soil over time. Moreover, S^2−^ may bind to humic substances and organic matter, potentially influencing redox dynamics in ways that further modulate N_2_O emissions (Heitmann and Blodau 2006). Additionally, SO_4_^2−^ materials are inexpensive and widely available, making them a practical choice for large‐scale field applications (Worthington et al. 2017). These properties make SO_4_^2−^ a more viable and sustainable candidate for mitigating N_2_O emissions in upland arable soils than Fe^3+^ and Mn^4+^.

### 4.2 Mechanism of Reducing N_2_O Emissions by MSs

In the heterogeneous environment of upland arable soils, we hypothesize that SO_4_^2−^ can function as an EA alongside NO_3_^−^ during anaerobic respiration. This simultaneous utilization of multiple EAs has the potential to minimize N_2_O emissions by altering the dynamics of EA competition and prioritization within microbial communities.

#### 4.2.1 Alterations in N‐Cycle Influenced by MS Application

The dual isotope mapping clearly demonstrated distinct shifts in SP values across treatments when plotted against δ^15^N^bulk^ − N_2_O and δ^18^O‐N_2_O (Figure 2). Specifically, the MS treatment exhibited significantly elevated SP values accompanied by pronounced depletion of δ^15^N^bulk^ relative to both the control and ZVM treatments, indicating a clear trend toward nitrification‐driven N_2_O production. This isotopic signature strongly suggests a fundamental redirection of electron flow within the soil N‐cycle under MS treatments. Such changes likely occurred due to increased competition for substrate electrons between SO_4_^2−^ reducers and denitrifying microbes. The electron demand of SO_4_^2−^ reduction presumably suppressed denitrification activity, thereby reducing the associated N_2_O emissions and shifting the overall balance of N_2_O production toward nitrification processes.

In contrast, the ZVM treatment showed isotopic signatures (both SP and δ^15^N^bulk^) that closely overlapped with those of the control soils, despite the observed increase in N_2_O emissions. Our initial interpretation, attributing higher N_2_O fluxes under ZVM solely to enhanced denitrification based on the result of 16S rRNA gene abundance (Figure 1b), was inconsistent with this isotopic evidence. To reconcile this discrepancy, we revised our interpretation, proposing that the electron‐donating properties of ZVM may have broadly stimulated microbial activities, enhancing both nitrification and denitrification pathways simultaneously. While denitrification is traditionally viewed as electron‐demanding, recent literature highlights that the early stages of nitrification also involve electron‐dependent activation of molecular oxygen. This initial electron requirement leads to the formation of hydroxylamine (Siegbahn 2024; Voss et al. 2024), an important intermediate and direct precursor of N_2_O. Thus, in ZVM‐treated soils, electron availability might have stimulated both nitrification and denitrification without strongly biasing the isotopic signatures toward either pathway, resulting in increased total N_2_O emissions but minimal changes in SP and δ^15^N^bulk^ values.

Collectively, our results underscore the complexity of interpreting isotopic data when multiple microbial processes coexist and are simultaneously influenced by treatment‐specific changes in electron availability. While MS clearly redirected electron flow toward sulfate reduction, thereby decreasing denitrification and emphasizing nitrification‐related N_2_O emissions, ZVM treatments enhanced microbial activity broadly without substantially shifting isotopic signatures. These nuanced observations emphasize that isotopic data interpretation must carefully consider simultaneous microbial processes and electron dynamics to accurately discern mechanisms underlying N_2_O production (Amundson et al. 2023).

The results for exchangeable ions supported the isotope mapping findings. MS application inhibited NO_3_^−^ reduction rather than NH_4_^+^ oxidation (Figure 3a,b). Notably, NO_3_^−^ and SO_4_^2−^ consumption patterns showed inverse relationships (Figure 3c,d). Compared to the control, NO_3_^−^ consumption was significantly reduced by approximately 10 mg kg^−1^ with MS application. Conversely, SO_4_^2−^ reduction in MS treatments averaged 66 mg kg^−1^, accounting for around 30% of the applied SO_4_^2−^, indicating SO_4_^2−^ likely outcompeted NO_3_^−^ as an EA under anaerobic conditions. Luo et al. (2022) reported decreased NO_3_^−^ reduction under elevated SO_4_^2−^ concentrations, with residual NO_3_^−^ ratios increasing by up to 60%. However, despite the large N input, the observed NO_3_^−^ consumption was unexpectedly about six times lower than that of SO_4_^2−^. This discrepancy is likely because NO_3_^−^ consumption was estimated from the difference between incubation days 21 and 7; potentially missing ongoing nitrification or intermediate N transformations occurring beyond the initial incubation period. Given the inherent complexity and continuous transformations within the soil N cycle, accurately quantifying exact NO_3_^−^ consumption presents a methodological challenge. Temporal changes in extractable NO_3_^−^ and NH_4_^+^ concentrations during incubation are provided in Figure S6.

Our study highlights the importance of further investigating the interactions between NO_3_^−^ and SO_4_^2−^ adsorption onto soils, which could significantly influence their bioavailability and microbial utilization, especially depending on soil pH (Essington and Stewart 2018; Zhou et al. 2025). Understanding how differing adsorption capacities of NO_3_^−^ and SO_4_^2−^ shape microbial competition and N cycling dynamics can yield valuable insights. Future research should incorporate detailed mineralogical and physicochemical analyses to enhance our knowledge, ultimately helping to optimize SO_4_^2−^ amendments for effectively mitigating N_2_O emissions across diverse soil conditions.

#### 4.2.2 Soil Microbial Quantity and Functional Gene Expression

Results from the functional gene expression analyses related to the N‐cycle (Figure 4) suggest concomitant activity of nitrification and denitrification and a significant decrease in the expression of the *nosZ* gene, which encodes the enzyme responsible for reducing N_2_O to N_2_. Such a decrease could potentially elevate N_2_O emissions by minimizing the efficiency of N_2_O reduction. However, a comprehensive evaluation indicated that NH_3_ oxidation remained unaffected. Other denitrification‐related genes (*nirK*, *nirS*, and *norB*) showed decreasing trends following MS application, but these changes were not statistically significant. Therefore, the substantial downregulation of *nosZ*, coupled with stable nitrification and minor decreases in other denitrification‐related genes, suggests an overall suppression of denitrification activity, resulting in reduced total N_2_O production. Consequently, despite the impaired potential for complete N_2_O reduction, total N_2_O emissions from MS‐treated soils remained lower compared to the control, predominantly due to the reduced overall N_2_O production through denitrification.

Furthermore, the abundance of *dsrA* mRNA was significantly increased under MS treatments, ranging from 8.14 × 10^4^ to 2.64 × 10^5^ copies per gram of dry soil (Figure S7). This increase in *dsrA* mRNA suggests that SO_4_^2−^, when added as an EA in MS treatments, stimulated the expression of genes encoding for the bisulfite reductase of SO_4_^2−^ reducers, thus indicating stimulation of active SO_4_^2−^ reduction as an anaerobic respiration process, potentially leading to decreased N_2_O emissions by outcompeting denitrification. Such observations align with findings from sulfate‐rich environments (e.g., coastal wetlands and riverbed soils) where NO_3_–^−^ consumption processes, such as denitrification and dissimilatory ^−−^NO3–/NO2 –reduction to NH_4_^+^+, are inhibited, decreasing N_2_O production (Bourceau et al. 2023; Cui et al. 2020). In the metabolic pathways of SOB, SO_4_^2−^ serves as a terminal EA, being reduced to sulfides (H_2_S, HS^−^, or S^2−^). Sulfides can inhibit N_2_O reduction, likely due to their toxicity and the formation of copper sulfides that interfere with key microbial enzymes (Pan et al. 2013). However, in well‐aerated arable soils, the large‐scale accumulation of toxic sulfides is unlikely. Under aerobic or fluctuating redox conditions, sulfur‐oxidizing microorganisms can re‐oxidize sulfides back to SO_4_^2−^. Furthermore, HS–^−^ and S^2−^ can precipitate with divalent metals such as Fe^2+^, forming insoluble FeS (Berkowitz et al. 2019). Although the overall risk of sustained sulfide toxicity is low, transient sulfide production under temporarily oxygen‐limited conditions could still disrupt denitrification by interfering with microbial activity or altering electron flow dynamics.

#### 4.2.3 Soil Microbial Community

Our study aims to identify materials that effectively reduce N_2_O emissions from soil. Thus, our microbial community analysis focused on MS treatments, which were effective in decreasing N_2_O emissions (Figure 1). Soils are known to harbor diverse microbial communities that play multifunctional roles in nutrient cycling, plant functional stability, and overall plant performance (Lou et al. 2022). In this study, although overall microbial diversity did not differ significantly among the treatments, MS supplementation led to an increased number of unique microbial genera (approximately 10% per treatment), suggesting treatment‐specific shifts in microbial community composition. Notably, a substantial proportion (~60%) of the total detected genera were minor taxa (relative abundances below 1%), highlighting the high complexity of the soil microbiota, in agreement with previous findings (Jiao et al. 2022; Jiao et al. 2019). Using ANCOM‐bc2 analysis, we identified 24 differentially abundant genera between the control and MS treatments, representing 9 abundant genera in the control and 15 abundant genera in the MS groups (Figure 7a and Table S4). In addition, Spearman correlation analysis revealed significant associations between key differentially abundant genera and various soil parameters and N cycling genes (Figure 7b).

*Nitrospira*, traditionally a known nitrifier, especially a nitrite‐oxidizing bacterium, was more abundant in the control than in MS (Figure 7a). Findings have identified comammox‐capable species that perform complete ammonia oxidation, expanding their recognized role in the N‐cycle (Daims et al. 2015). While *Nitrospira* is not directly involved in denitrification or N_2_O reduction, its contribution to upstream nitrification may indirectly influence N_2_O emissions, particularly in C‐rich microzones where redox fluctuations facilitate tight nitrification–denitrification coupling (Kits et al. 2019). In our study, suppressing *Nitrospira* under MS treatments could reflect altered redox or substrate conditions unfavorable to nitrification. These shifts may indirectly influence N_2_O emissions by disrupting the continuity of nitrification–denitrification coupling. *UG_Gemmatimonadaceae*, *Herminiimonas*, and *Ureibacillus* were enriched in sulfate‐treated soils and showed negative correlations with *nosZ* (Figure 7b), suggesting potential shifts in redox dynamics and microbial functional activity. Members of the family *Gemmatimonadaceae*, belonging to the phylum *Gemmatimonadota*, are widely distributed across diverse environments such as soils, sediments, and marine (Fudjoe et al. 2023; Gong et al. 2024). Recent metagenomic studies have revealed that *Gemmatimonadaceae* possesses genes related to SO_4_^2−^ reduction but lacks genetic machinery for denitrification (Mujakić et al. 2023). Similarly, Koh et al. (2017) reported that *Herminiimonas arsenitoxidans AS8* harbors genes associated with SO_4_^2−^ reduction yet does not contain denitrification‐related genes. In contrast, *Ureibacillus thermosphaerius strain thermos‐BF*, as reported by Juibari et al. (2015), contains both genes of denitrification and SO_4_^2−^ reduction, highlighting its dual metabolic capabilities.

Metal sulfate treatments specifically enriched various sulfur‐cycling bacteria taxa, including *Magnetospirillaceae*, *UCG_Rhodospirillales*, and *Afipia* (Figure 7a). Previous studies have reported that certain members of *Magnetospirillaceae* and *UCG_Rhodospirillales* (both belonging to the order *Rhodospirillales*) participate in sulfide oxidation and thus function as sulfur‐oxidizing bacteria (SOB) (Koziaeva et al. 2023; Yang et al. 2023). *Afipia* is also known for its capacity to oxidize reduced sulfur compounds (Huddy et al. 2021). The observed increase in SRB under MS treatments likely enhanced sulfide production, subsequently stimulating SOB. This increase in SOB activity could potentially suppress denitrification efficiency, leading to a reduction in overall N_2_O emissions (Lu et al. 2018). Supporting this, we identified significant positive correlations between specific microbial genera and both SO_4_^2−^ concentrations and *dsrA* expression, indicating enhanced SO_4_^2−^ utilization and *dsrA* expression following MS supplementation. For instance, *AKYG1722*, which was notably higher in relative abundance under MS treatments, is known for utilizing SO_4_^2−^ in N‐depleted environments (González‐Cortés et al. 2022). Introducing SO_4_^2−^ into the soil markedly decreased the relative abundance of denitrification‐related microorganisms, such as *Elsterales* and *Anaeromyxobacter* (Mhuantong et al. 2015; Zecchin et al. 2023). This change in the microbial community, particularly in soils with high SO_4_^2−^ concentrations, likely contributed to decreased NO_3_^−^ consumption. The relationship between SO_4_^2−^ and NO_3_^−^ respiration may be more direct, as many organisms traditionally identified as SRB can also reduce NO_2_^−^ through denitrification or DNRA (Pan et al. 2013). Some organisms transition to NO_3_^−^/NO_2_^−^ respiration when N‐oxides are accessible (Krekeler and Cypionka 1995; Seitz and Cypionka 1986), while others consistently prioritize SO_4_^2−^ reduction even when oxidized N compounds are present (Dalsgaard and Bak 1994; Marietou 2016; Marietou et al. 2009).

### 4.3 Feasibility of MSs for Mitigating N_2_O Emissions in Upland Arable Soils

The mean MEY over the 2 years did not differ significantly between the control and MS treatments (Table 1). Mahal et al. (2022) noted no increase in maize production at half of the 12 sites studied despite using four S fertilizers, indicating a weak positive relationship between corn productivity and S application. Although MnSO_4_ showed an increase in MEY compared to the control in Y1, this does not conclusively indicate that MS can enhance crop productivity. A critical aspect of our field experiment was to evaluate the feasibility of SO_4_^2−^ materials as soil amendments. Our results demonstrated that applying SO_4_^2−^ at 20 kg ha^−1^ of MS did not adversely affect crops. Other studies have shown that a S input of 15–50 kg ha^−1^ can enhance pest resistance, tissue strength, and crop quality. In maize cultivation fields, YSNE values have been reported to be above 1 kg Mg^−1^ (Halvorson et al. 2010), but recent global meta‐analyses have indicated a range of 0.18–0.36 kg Mg^−1^ (Aliyu et al. 2021). YSNE is influenced by factors such as irrigation, temperature, and initial total N concentration (Wang et al. 2021). There is a strong correlation between NF input and YSNE (Kim et al. 2023). Given the importance of food security, unconditionally reducing NF input is challenging. Additionally, crop N uptake is closely linked to sulfur metabolism; our measurements showed no significant differences in total N uptake between the control and MS treatments (Table S5). Thus, enhanced N uptake does not appear to explain the observed mitigation in N_2_O emissions in our study. Therefore, SO_4_^2−^ should be considered a potential fertilizer or soil amendment for its ability to mitigate N_2_O emissions in arable soils, balancing environmental concerns with agricultural productivity.

Changes in soil physicochemical properties following MS application can be indirectly assessed through soil pH variations. The applied MS treatments led to a minor and statistically non‐significant decline in soil pH throughout the cultivation period (Figure S8). Although metal ions such as Fe^2+^, Mn^2+^, and Zn^2+^ can release protons (H^+^) during oxidation and contribute to soil acidification (Grundl and Delwiche 1993; Lindsay 1979a; Menard and Demopoulos 2007; Permyakov 2021; Schwertmann and Murad 1983), the relatively low application rates used here resulted in minimal and insignificant changes. Furthermore, intermittent SO_4_^2−^ reduction and S^2−^ oxidation processes, typical of partially anaerobic microsites in upland arable soils, likely buffered these acidification effects. Despite this limited pH response, even subtle pH changes could influence microbial communities involved in nitrification and denitrification processes, potentially affecting ammonia oxidizer and denitrifier activities (Shaaban et al. 2018; Zhou et al. 2024). Thus, minor soil pH alterations induced by MS applications might partially contribute to the observed N_2_O mitigation effects, although this is likely a secondary mechanism.

Furthermore, SO_4_^2−^ applications have implications beyond N cycling, significantly influencing additional biogeochemical processes in agroecosystems such as the C cycle. Microbial SO_4_^2−^ reduction may initially stimulate CO_2_ emissions due to enhanced organic matter mineralization. However, the subsequent formation of sulfide compounds introduces additional complexity. For example, Pan et al. (2013) demonstrated that sulfide compounds formed through SO_4_^2−^ reduction could inhibit microbial respiration, potentially decreasing CO_2_ emissions from soils. Additionally, Wu et al. (2021) suggested that these sulfide compounds may chemically interact with soil organic matter, influencing its long‐term stability and overall carbon sequestration potential. These complex interactions underscore the multifaceted influence of microbial S cycling on other elemental cycles and highlight the challenge of accurately predicting long‐term impacts on soil C dynamics and greenhouse gas emissions.

The potential for SO_4_^2−^ reduction in upland arable soils fundamentally differs from conditions typically encountered in marine sediments, wetlands, or rice paddy soils, where prolonged anoxic conditions enable continuous SO_4_^2−^ reduction. In contrast, upland agricultural soils develop transient anaerobic microsites due to events such as heavy rainfall, intensive irrigation, or specific management practices, creating fluctuating soil redox conditions that alternate frequently between oxidized SO_4_^2−^ and reduced S^2−^ forms. Nevertheless, our experimental results provide evidence that SO_4_^2−^ reduction can still transiently occur under specific conditions in upland arable soils. This observation aligns with earlier studies, particularly those by Canfield and Des Marais (1991), who reported dissimilatory SO_4_^2−^ reduction even in partially oxygenated environments. Such findings challenge the conventional assumption that SO_4_^2−^ reduction is exclusively an anaerobic process and highlight the necessity for further research to better characterize and understand the specific environmental conditions that permit SO_4_^2−^ reduction under partially aerobic circumstances.

### 4.4 Literature Review on the Role of Sulfate in Mitigating N_2_O Emissions

**FIGURE 8:**
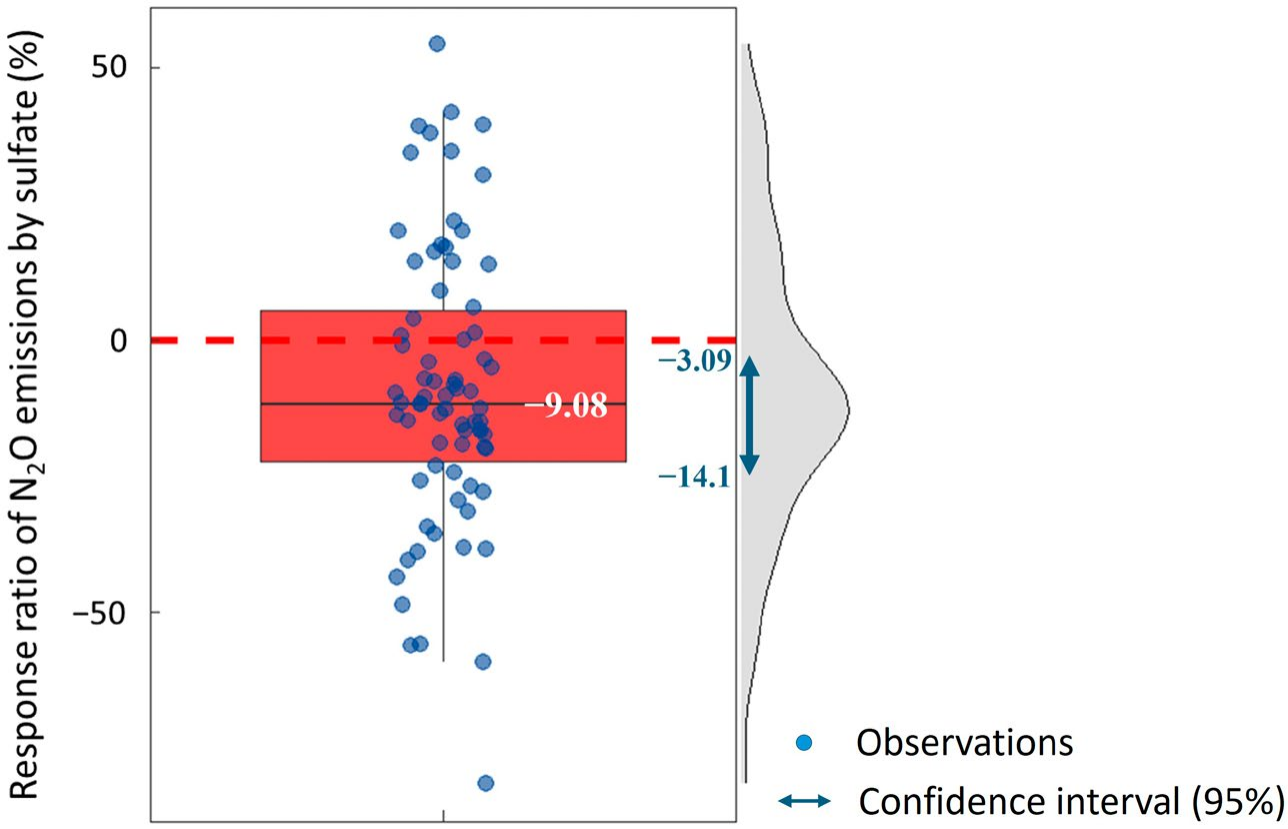
Response ratio of N_2_O emissions to sulfate addition across globally selected sites. This plot presents data compiled through a PRISMA‐based systematic literature review. Each blue dot (*N* = 76) represents an individual observation comparing sulfate‐treated soils to conventional management practices. The red boxplot summarizes the distribution of response ratios, with the solid black line indicating the mean (−9.08%) and the red dashed lines showing the 95% confidence interval (CI), which ranges from −14.10% to −3.09%. Since the CI does not include zero, this confirms a statistically significant reduction in N_2_O emissions. The accompanying violin plot illustrates the distribution density of the effect sizes.

Although the findings from this study support our hypothesis, they challenge the conventional understanding of alternative EA usage, which may raise critical questions about generalizing these results. To broaden the scope of this hypothesis, we conducted a systematic literature review (Table S6) and a global meta‐analysis to explore the effects of SO_4_^2−^ additions on N_2_O emissions in cultivated aerobic condition soils, particularly upland arable soils. The response ratio was calculated by analyzing the effects of SO_4_^2−^ addition relative to control treatments without SO_4_^2−^ addition. The dataset covered diverse SO_4_^2−^ application rates, crop types, and soil conditions, strengthening the findings' relevance and applicability across different agricultural scenarios. Our results demonstrate that SO_4_^2−^ amendment significantly decreases N_2_O emissions, with a mean response ratio of −9.08% (SD = 28.4%, *N* = 76) and a 95% CI ranging from −14.10% to −3.09% (Figure 8). Importantly, as the 95% CI does not include zero, this minimization is statistically significant. Among the 76 individual observations analyzed, 70.3% exhibited negative response ratios, further supporting the effectiveness of SO_4_^2−^ amendments in mitigating N_2_O emissions from upland arable soils. These findings align well with our field and microcosm experiments, reinforcing the potential of SO_4_^2−^ as a viable alternative EA. Additionally, SO_4_^2−^ can function as an EA even under micro‐oxic conditions, not requiring fully anoxic environments like Fe^3+^ and CO_2_ reduction (Brodersen et al. 2019). The results suggest that SO_4_^2−^ amendment is an effective and broadly applicable strategy for reducing N_2_O emissions from upland arable soils.

Our study used MSs, specifically FeSO_4_, MnSO_4_, and ZnSO_4_, to evaluate their potential as alternative EAs. However, the existing literature shows that other sulfate‐based materials, such as CaSO_4_ and MgSO_4_, are frequently used in similar studies. These SO_4_^2−^ forms, including by‐products such as phosphogypsum, desulfurization gypsum, and blast furnace slag, are readily available and cost‐effective EAs and provide essential macronutrients (Lee et al. 2023). Additionally, our study demonstrates that applying MS at a moderate rate of 20 kg ha^−1^ can significantly reduce N_2_O emissions. However, a review of existing literature reveals substantial variability in application rates and outcomes reported across different studies, highlighting an urgent need for further exploration. For instance, Gao et al. (2024) found effective N_2_O mitigation with a high gypsum application rate of approximately 1.44 Mg ha^−1^ (converted from 0.72 g kg^−1^ of soil); whereas Watts et al. (2023) reported no significant effects even with a higher application rate of 2.20 Mg ha^−1^. On the other hand, much lower SO_4_^2−^ application rates of 10–27.5 kg ha^−1^ have also been reported to effectively reduce N_2_O emissions in maize cultivation fields (Majumdar et al. 2002; Montoya et al. 2021). The inconsistencies in these findings likely result from variations in SO_4_^2−^ forms, along with differing soil properties, crop management practices, microbial community dynamics, and local climate conditions. Thus, it is imperative that future research comprehensively examines these factors. By doing so, we can gain deeper insights into the complex interactions that determine the effectiveness of SO_4_^2−^ in mitigating N_2_O emissions from arable soils, paving the way for more effective agricultural practices.

## 5 Summary

Validating SO_4_^2−^ material as an effective soil amendment for reducing N_2_O emissions in upland arable soils hinges on two critical aspects emphasized in this study. First, it is essential to determine whether alternative EAs can be utilized under partially anaerobic conditions created by rainfall and irrigation in upland arable soils. Second, it is crucial to evaluate whether SO_4_^2−^, despite being a lower‐priority EA compared to NO_3_^−^, can be used concurrently with NO_3_^−^.

The results of this study, particularly from isotope mapping, NO_3_^−^ and SO_4_^2−^ measurements, and gene expression, confirmed that SO_4_^2−^ treatments significantly suppressed N_2_O production from denitrification and stimulated SO_4_^2−^ reduction. The results of metataxonomic analysis supported these findings, showing an increase in microbial taxa capable of using both NO_3_^−^ and SO_4_^2−^ as EAs in MS‐treated soils. Furthermore, MS treatments enriched sulfur‐cycling microbial taxa and concurrently suppressed nitrifiers. This microbial community shift suggests that the concurrent utilization of NO_3_^−^ and SO_4_^2−^ as EAs and the suppression of nitrifiers may have disrupted the coupling between nitrification and denitrification, thereby contributing to the reduction in N_2_O emissions. Field‐manipulation experiments further demonstrated that adding practical amounts of MSs to soils effectively reduced YSNE. Additionally, due to greater bioavailability under less stringent redox conditions, SO_4_^2−^ offers a more practical alternative EA than Fe^3+^ and Mn^4+^ in upland arable soils. This unique characteristic of SO_4_^2−^ reduction opens up a new avenue for research and application, as it can be utilized alongside NO_3_^−^ under partially anoxic conditions, highlighting the potential of SO_4_^2−^ materials as a viable and intriguing soil amendment that promotes sustainable agriculture practices while mitigating GHG emissions.

## Author Contributions

**Hyun Ho Lee:** conceptualization, data curation, formal analysis, funding acquisition, investigation, methodology, supervision, validation, visualization, writing – original draft, writing – review and editing. **Hanbeen Kim:** data curation, formal analysis, visualization, writing – original draft, writing – review and editing. **Ye Lim Park:** formal analysis, investigation. **Marcus A. Horn:** supervision. **Jeongeun Kim:** formal analysis, investigation, methodology, visualization. **Jaehyun Lee:** investigation, visualization. **Sakae Toyoda:** investigation, methodology, visualization. **Jeongeun Yun:** formal analysis, investigation. **Hojeong Kang:** methodology, resources. **Sang Yoon Kim:** formal analysis. **Jinho Ahn:** formal analysis. **Chang Oh Hong:** conceptualization, funding acquisition, project administration, supervision.

## Conflicts of Interest

The authors declare no conflicts of interest.

## Supporting information


**Data S1:** gcb70428‐sup‐0001‐supinfo.zip.

## Data Availability

The data and code that support the findings of this study are openly available in Zenodo at https://doi.org/10.5281/zenodo.16389568, and in the NCBI Sequence Read Archive (SRA) under BioProject number PRJNA1107391, with SRA accession numbers SRX24441768‐SRX24441776.

## References

[gcb70428-bib-0001] AAPFCO . 2012. AAPFCO Product Label Guide A. o. A. P. F. C. Officials.

[gcb70428-bib-0002] Aliyu, G. , L. Jiafa , L. Deyan , et al. 2021. “Yield‐Scaled Nitrous Oxide Emissions From Nitrogen‐Fertilized Croplands in China: A Meta‐Analysis of Contrasting Mitigation Scenarios.” Pedosphere 31, no. 2: 231–242.

[gcb70428-bib-0003] Amissah, S. , G. Ankomah , R. D. Lee , et al. 2024. “Secondary and Micronutrient Application Effects on Corn Fertilized for Different Yield Goals Under Highly Weathered Soil Conditions.” Agronomy Journal 116, no. 2: 737–752.

[gcb70428-bib-0004] Amundson, R. , J. V. Mills , L. N. Lammers , et al. 2023. “Simultaneous Production and Consumption of Soil N_2_O Creates Complex Effects on Its Stable Isotope Composition.” Global Biogeochemical Cycles 37, no. 9: e2022GB007536.

[gcb70428-bib-0005] Becker, M. , and F. Asch . 2005. “Iron Toxicity in Rice—Conditions and Management Concepts.” Journal of Plant Nutrition and Soil Science 168, no. 4: 558–573.

[gcb70428-bib-0006] Berkowitz, J. F. , C. M. VanZomeren , and N. D. Fresard . 2019. “Rapid Formation of Iron Sulfides Alters Soil Morphology and Chemistry Following Simulated Marsh Restoration.” Geoderma 351: 76–84.

[gcb70428-bib-0007] Bethke, C. M. , R. A. Sanford , M. F. Kirk , Q. Jin , and T. M. Flynn . 2011. “The Thermodynamic Ladder in Geomicrobiology.” American Journal of Science 311, no. 3: 183–210.

[gcb70428-bib-0008] Bloem, E. , S. Haneklaus , and E. Schnug . 2002. “Optimization of a Method for Soil Sulphur Extraction.” Communications in Soil Science and Plant Analysis 33, no. 1–2: 41–51.

[gcb70428-bib-0009] Bolyen, E. , J. R. Rideout , M. R. Dillon , et al. 2019. “Reproducible, Interactive, Scalable and Extensible Microbiome Data Science Using QIIME 2.” Nature Biotechnology 37, no. 8: 852–857. 10.1038/s41587-019-0209-9 PMC7015180 31341288

[gcb70428-bib-0010] Bourceau, O. , T. Ferdelman , G. Lavik , M. Mussmann , M. Kuypers , and H. Marchant . 2023. “Simultaneous Sulfate and Nitrate Reduction in Coastal Sediments.” ISME Communications 3, no. 1: 17. 36882570 10.1038/s43705-023-00222-y PMC9992702

[gcb70428-bib-0011] Braker, G. , and R. Conrad . 2011. “Diversity, Structure, and Size of N_2_O‐Producing Microbial Communities in Soils—What Matters for Their Functioning?” Advances in Applied Microbiology 75: 33–70. 21807245 10.1016/B978-0-12-387046-9.00002-5

[gcb70428-bib-0012] Brodersen, K. E. , S. M. Trevathan‐Tackett , D. A. Nielsen , et al. 2019. “Oxygen Consumption and Sulfate Reduction in Vegetated Coastal Habitats: Effects of Physical Disturbance.” Frontiers in Marine Science 6: 14.

[gcb70428-bib-0013] Butterbach‐Bahl, K. , E. M. Baggs , M. Dannenmann , R. Kiese , and S. Zechmeister‐Boltenstern . 2013. “Nitrous Oxide Emissions From Soils: How Well Do We Understand the Processes and Their Controls?” Philosophical Transactions of the Royal Society, B: Biological Sciences 368, no. 1621: 20130122. 10.1098/rstb.2013.0122 PMC3682742 23713120

[gcb70428-bib-0014] Canfield, D. E. , and D. J. Des Marais . 1991. “Aerobic Sulfate Reduction in Microbial Mats.” Science 251, no. 5000: 1471–1473. 11538266 10.1126/science.11538266

[gcb70428-bib-0015] Canfield, D. E. , B. B. Jørgensen , H. Fossing , et al. 1993. “Pathways of Organic Carbon Oxidation in Three Continental Margin Sediments.” Marine Geology 113, no. 1–2: 27–40. 11539842 10.1016/0025-3227(93)90147-n

[gcb70428-bib-0016] Charlet, L. , N. Dise , and W. Stumm . 1993. “Sulfate Adsorption on a Variable Charge Soil and on Reference Minerals.” Agriculture, Ecosystems & Environment 47, no. 2: 87–102.

[gcb70428-bib-0017] Choi, E. J. H. , S. E. Kim , O. J. Joo , N. G. Kang , and H. O. Lee . 2023. “A Guide to Nitrous Oxide Measurement in Agricultural Soils for Novice Researchers—Focusing on the Chamber Method [in Korean].” National Institute of Agricultural Sciences, Rural Development Administration.

[gcb70428-bib-0018] Cui, L. , B. Zhu , X. Zhang , et al. 2020. “Influences of Organic Nitrogen on the Removal of Inorganic Nitrogen From Complicated Marine Aquaculture Water by *Marichromatium gracile* YL28.” Journal of Bioscience and Bioengineering 130, no. 2: 179–186. 32381439 10.1016/j.jbiosc.2020.02.016

[gcb70428-bib-0019] Daims, H. , E. V. Lebedeva , P. Pjevac , et al. 2015. “Complete Nitrification by Nitrospira Bacteria.” Nature 528, no. 7583: 504–509. 26610024 10.1038/nature16461 PMC5152751

[gcb70428-bib-0020] Dalsgaard, T. , and F. Bak . 1994. “Nitrate Reduction in a Sulfate‐Reducing Bacterium, *Desulfovibrio desulfuricans* , Isolated From Rice Paddy Soil: Sulfide Inhibition, Kinetics, and Regulation.” Applied and Environmental Microbiology 60, no. 1: 291–297. 16349159 10.1128/aem.60.1.291-297.1994 PMC201302

[gcb70428-bib-0021] Davidson, E. A. 2009. “The Contribution of Manure and Fertilizer Nitrogen to Atmospheric Nitrous Oxide Since 1860.” Nature Geoscience 2, no. 9: 659–662.

[gcb70428-bib-0022] Decock, C. , and J. Six . 2013. “How Reliable Is the Intramolecular Distribution of 15N in N_2_O to Source Partition N_2_O Emitted From Soil?” Soil Biology and Biochemistry 65: 114–127.

[gcb70428-bib-0023] Dhaliwal, S. , R. Naresh , A. Mandal , R. Singh , and M. Dhaliwal . 2019. “Dynamics and Transformations of Micronutrients in Agricultural Soils as Influenced by Organic Matter Build‐Up: A Review.” Environmental and Sustainability Indicators 1: 100007.

[gcb70428-bib-0024] Dong, D. , W. Yang , H. Sun , S. Kong , and H. Xu . 2022. “Effects of Split Application of Urea on Greenhouse Gas and Ammonia Emissions From a Rainfed Maize Field in Northeast China.” Frontiers in Environmental Science 9: 743.

[gcb70428-bib-0025] Eimers, M. C. , P. J. Dillon , S. L. Schiff , and D. S. Jeffries . 2003. “The Effects of Drying and Re‐Wetting and Increased Temperature on Sulphate Release From Upland and Wetland Material.” Soil Biology and Biochemistry 35, no. 12: 1663–1673.

[gcb70428-bib-0026] Epstein, E. , and A. J. Bloom . 2005. Mineral Nutrition of Plants: Principles and Perspectives. 2nd ed. Sinauer Associates, Inc.

[gcb70428-bib-0027] Essington, M. E. , and M. A. Stewart . 2018. “Adsorption of Antimonate, Sulfate, and Phosphate by Goethite: Reversibility and Competitive Effects.” Soil Science Society of America Journal 82, no. 4: 803–814.

[gcb70428-bib-0028] FAO . 2021. “Emissions due to Agriculture.” Global, Regional and Country Trends 2000–2018.

[gcb70428-bib-0029] Ferland, D. , C. Wagner‐Riddle , S. Brown , et al. 2024. “Improved Nitrogen Fertilizer Management Reduces Nitrous Oxide Emissions in a Northern Prairie Cropland.” Science of the Total Environment 956: 177211. 39481573 10.1016/j.scitotenv.2024.177211

[gcb70428-bib-0030] Freeman, C. G. 2020. Upland Flood Mitigation by Managing Infiltration Aberystwyth University.

[gcb70428-bib-0031] Fudjoe, S. K. , L. Li , S. Anwar , et al. 2023. “Nitrogen Fertilization Promoted Microbial Growth and N_2_O Emissions by Increasing the Abundance of *nirS* and *nosZ* Denitrifiers in Semiarid Maize Field.” Frontiers in Microbiology 14: 1265562. 37720157 10.3389/fmicb.2023.1265562 PMC10501401

[gcb70428-bib-0032] Galloway, J. N. , A. Bleeker , and J. W. Erisman . 2021. “The Human Creation and Use of Reactive Nitrogen: A Global and Regional Perspective.” Annual Review of Environment and Resources 46, no. 1: 255–288.

[gcb70428-bib-0033] Gao, P. , X. Yan , X. Xia , et al. 2024. “Effects of the Three Amendments on NH_3_ Volatilization, N_2_O Emissions, and Nitrification at Four Salinity Levels: An Indoor Experiment.” Journal of Environmental Management 354: 120399. 38387357 10.1016/j.jenvman.2024.120399

[gcb70428-bib-0034] Gong, X. , L. Xu , M. V. Langwig , et al. 2024. “Globally Distributed Marine Gemmatimonadota Have Unique Genomic Potentials.” Microbiome 12, no. 1: 149. 39123272 10.1186/s40168-024-01871-4 PMC11316326

[gcb70428-bib-0035] González‐Cortés, J. J. , A. Valle , M. Ramírez , and D. Cantero . 2022. “Characterization of Bacterial and Archaeal Communities by DGGE and Next Generation Sequencing (NGS) of Nitrification Bioreactors Using Two Different Intermediate Landfill Leachates as Ammonium Substrate.” Waste and Biomass Valorization 13, no. 9: 3753–3766.

[gcb70428-bib-0036] Govil, S. , N. V. D. Long , M. Escribà‐Gelonch , and V. Hessel . 2024. “Controlled‐Release Fertiliser: Recent Developments and Perspectives.” Industrial Crops and Products 219: 119160.

[gcb70428-bib-0037] Griffiths, R. I. , A. S. Whiteley , A. G. O'Donnell , and M. J. Bailey . 2000. “Rapid Method for Coextraction of DNA and RNA From Natural Environments for Analysis of Ribosomal DNA‐ and rRNA‐Based Microbial Community Composition.” Applied and Environmental Microbiology 66, no. 12: 5488–5491. 11097934 10.1128/aem.66.12.5488-5491.2000 PMC92488

[gcb70428-bib-0038] Grundl, T. , and J. Delwiche . 1993. “Kinetics of Ferric Oxyhydroxide Precipitation.” Journal of Contaminant Hydrology 14, no. 1: 71–87.

[gcb70428-bib-0039] Gupta, U. C. , and S. C. Gupta . 1998. “Trace Element Toxicity Relationships to Crop Production and Livestock and Human Health: Implications for Management.” Communications in Soil Science and Plant Analysis 29, no. 11–14: 1491–1522.

[gcb70428-bib-0040] Hafeez, M. B. , Y. Ramzan , S. Khan , et al. 2021. “Application of Zinc and Iron‐Based Fertilizers Improves the Growth Attributes, Productivity, and Grain Quality of Two Wheat (*Triticum aestivum*) Cultivars.” Frontiers in Nutrition 8: 779595. 34966772 10.3389/fnut.2021.779595 PMC8710766

[gcb70428-bib-0041] Halvorson, A. D. , S. J. Del Grosso , and F. Alluvione . 2010. “Nitrogen Source Effects on Nitrous Oxide Emissions From Irrigated no‐Till Corn.” Journal of Environmental Quality 39, no. 5: 1554–1562. 10.2134/jeq2010.0041. 21043261

[gcb70428-bib-0042] Heitmann, T. , and C. Blodau . 2006. “Oxidation and Incorporation of Hydrogen Sulfide by Dissolved Organic Matter.” Chemical Geology 235, no. 1–2: 12–20.

[gcb70428-bib-0043] Hem, J. D. 1963. Chemical Equilibria and Rates of Manganese Oxidation. US Government printing office.

[gcb70428-bib-0044] Hu, L. , Z. Dong , Z. Wang , L. Xiao , and B. Zhu . 2022. “The Contributions of Ammonia Oxidizing Bacteria and Archaea to Nitrification‐Dependent N_2_O Emission in Alkaline and Neutral Purple Soils.” Scientific Reports 12, no. 1: 19928. 36402873 10.1038/s41598-022-23084-1 PMC9675842

[gcb70428-bib-0045] Huang, T. , H. Yang , C. Huang , and X. Ju . 2017. “Effect of Fertilizer N Rates and Straw Management on Yield‐Scaled Nitrous Oxide Emissions in a Maize‐Wheat Double Cropping System.” Field Crops Research 204: 1–11.

[gcb70428-bib-0046] Huddy, R. J. , R. Sachdeva , F. Kadzinga , R. S. Kantor , S. T. Harrison , and J. F. Banfield . 2021. “Thiocyanate and Organic Carbon Inputs Drive Convergent Selection for Specific Autotrophic Afipia and Thiobacillus Strains Within Complex Microbiomes.” Frontiers in Microbiology 12: 643368. 33897653 10.3389/fmicb.2021.643368 PMC8061750

[gcb70428-bib-0047] Ibraim, E. , T. Denk , B. Wolf , et al. 2020. “Denitrification Is the Main Nitrous Oxide Source Process in Grassland Soils According to Quasi‐Continuous Isotopocule Analysis and Biogeochemical Modeling.” Global Biogeochemical Cycles 34, no. 6: e2019GB006505.

[gcb70428-bib-0048] Inatomi, M. , T. Hajima , and A. Ito . 2019. “Fraction of Nitrous Oxide Production in Nitrification and Its Effect on Total Soil Emission: A Meta‐Analysis and Global‐Scale Sensitivity Analysis Using a Process‐Based Model.” PLoS One 14, no. 7: e0219159. 31291317 10.1371/journal.pone.0219159 PMC6619742

[gcb70428-bib-0049] IPCC . 2019. “Chapter 11: N_2_O and CO_2_ Emissions From Managed Soils, and CO_2_ Emissions From Lime and Urea Application (Volume 4, Chapter 11).” 2019 Refinement to the 2006 IPCC Guidelines for National Greenhouse Gas Inventories, Volume 4: Agriculture, Forestry and Other Land Use, Issue. https://www.ipcc‐nggip.iges.or.jp/public/2019rf/pdf/4_Volume4/19R_V4_Ch11_Soils_N2O_CO2.pdf.

[gcb70428-bib-0050] Jakobsen, R. , and D. Postma . 1999. “Redox Zoning, Rates of Sulfate Reduction and Interactions With Fe‐Reduction and Methanogenesis in a Shallow Sandy Aquifer, Rømø, Denmark.” Geochimica et Cosmochimica Acta 63, no. 1: 137–151.

[gcb70428-bib-0051] Jiao, S. , W. Chen , and G. Wei . 2022. “Core Microbiota Drive Functional Stability of Soil Microbiome in Reforestation Ecosystems.” Global Change Biology 28, no. 3: 1038–1047. 34862696 10.1111/gcb.16024

[gcb70428-bib-0052] Jiao, S. , Y. Xu , J. Zhang , X. Hao , and Y. Lu . 2019. “Core Microbiota in Agricultural Soils and Their Potential Associations With Nutrient Cycling.” MSystems 4, no. 2: 10‐1128. 10.1128/msystems.00313-00318. PMC6435817 30944882

[gcb70428-bib-0053] Jørgensen, B. , A. Findlay , and A. Pellerin . 2019. “The Biogeochemical Sulfur Cycle of Marine Sediments.” Frontiers in Microbiology 10: 849. 31105660 10.3389/fmicb.2019.00849 PMC6492693

[gcb70428-bib-0054] Juibari, M. M. , L. P. Yeganeh , S. Abbasalizadeh , et al. 2015. “Investigation of a Hot‐Spring Extremophilic *Ureibacillus thermosphaericus* Strain Thermo‐BF for Extracellular Biosynthesis of Functionalized Gold Nanoparticles.” BioNanoScience 5: 233–241.

[gcb70428-bib-0055] Kim, D.‐G. , D. Giltrap , and T. B. Sapkota . 2023. “Understanding Response of Yield‐Scaled N_2_O Emissions to Nitrogen Input: Data Synthesis and Introducing New Concepts of Background Yield‐Scaled N_2_O Emissions and N_2_O Emission‐Yield Curve.” Field Crops Research 290: 108737.

[gcb70428-bib-0056] Kits, K. D. , M.‐Y. Jung , J. Vierheilig , et al. 2019. “Low Yield and Abiotic Origin of N_2_O Formed by the Complete Nitrifier Nitrospira Inopinata.” Nature Communications 10, no. 1: 1836. 10.1038/s41467-019-09790-x PMC6478695 31015413

[gcb70428-bib-0057] Klimczyk, M. , A. Siczek , and L. Schimmelpfennig . 2021. “Improving the Efficiency of Urea‐Based Fertilization Leading to Reduction in Ammonia Emission.” Science of the Total Environment 771: 145483. 33736136 10.1016/j.scitotenv.2021.145483

[gcb70428-bib-0058] Klüber, H. D. , and R. Conrad . 1998. “Effects of Nitrate, Nitrite, NO and N_2_O on Methanogenesis and Other Redox Processes in Anoxic Rice Field Soil.” FEMS Microbiology Ecology 25, no. 3: 301–318. 10.1111/j.1574-6941.1998.tb00482.x.

[gcb70428-bib-0059] Kodithuwakku, K. , J. Huang , C. L. Doolette , et al. 2024. “Plant Responses to Nitrate and Ammonium Availability in Australian Soils as Measured by Diffusive Gradients in Thin‐Films (DGT) and KCl Extraction.” Geoderma 449: 116997.

[gcb70428-bib-0060] Koh, H.‐W. , M. Hur , M.‐S. Kang , Y.‐B. Ku , R. Ghai , and S.‐J. Park . 2017. “Physiological and Genomic Insights Into the Lifestyle of Arsenite‐Oxidizing Herminiimonas Arsenitoxidans.” Scientific Reports 7, no. 1: 15007. 29101383 10.1038/s41598-017-15164-4 PMC5670224

[gcb70428-bib-0061] Korea Meteorological Administration . 2025. “Automated Synoptic vObserving System (ASOS) Data.” https://data.kma.go.kr.

[gcb70428-bib-0062] Koziaeva, V. V. , D. Y. Sorokin , T. V. Kolganova , and D. S. Grouzdev . 2023. “Magnetospirillum Sulfuroxidans sp. Nov., Capable of Sulfur‐Dependent Lithoautotrophy and a Taxonomic Reevaluation of the Order Rhodospirillales.” Systematic and Applied Microbiology 46, no. 3: 126406. 36898262 10.1016/j.syapm.2023.126406

[gcb70428-bib-0063] Krekeler, D. , and H. Cypionka . 1995. “The Preferred Electron Acceptor of *Desulfovibrio desulfuricans* CSN.” FEMS Microbiology Ecology 17, no. 4: 271–277.

[gcb70428-bib-0064] Lahti, L. , J. Salojärvi , A. Salonen , M. Scheffer , and W. M. De Vos . 2014. “Tipping Elements in the Human Intestinal Ecosystem.” Nature Communications 5, no. 1: 4344. 10.1038/ncomms5344 PMC4102116 25003530

[gcb70428-bib-0065] Lee, H. H. , Y. D. Noh , S. Park , et al. 2023. “Optimizing Calcium Materials for Minimizing Arsenate Phytoavailability in Upland Arable Soil Based on Geochemical Analysis.” Journal of Hazardous Materials 448: 130927. 36764253 10.1016/j.jhazmat.2023.130927

[gcb70428-bib-0066] Lin, H. , and S. D. Peddada . 2020. “Analysis of Compositions of Microbiomes With Bias Correction.” Nature Communications 11, no. 1: 3514. 10.1038/s41467-020-17041-7 PMC7360769 32665548

[gcb70428-bib-0067] Lindsay, W. 1979a. Chemical Equilibria in Soils. John Willey & Sons.

[gcb70428-bib-0068] Lindsay, W. L. 1979b. Chemical Equilibria in Soils. John Wiley and Sons Ltd.

[gcb70428-bib-0069] Liu, X. , Z. Yan , C. Wu , Y. Yang , X. Li , and G. Zhang . 2019. “FastProNGS: Fast Preprocessing of Next‐Generation Sequencing Reads.” BMC Bioinformatics 20: 1–6. 31208325 10.1186/s12859-019-2936-9 PMC6580563

[gcb70428-bib-0070] Lou, J. , H. Jin , J. Li , J. Lv , F. Xu , and R. Wang . 2022. “The Mechanism of Sulfate on a Nitrate Denitrifying Anaerobic Methane Oxidation System.” Environmental Science: Water Research & Technology 8, no. 12: 2884–2894.

[gcb70428-bib-0071] Lu, H. , H. Huang , W. Yang , et al. 2018. “Elucidating the Stimulatory and Inhibitory Effects of Dissolved Sulfide on Sulfur‐Oxidizing Bacteria (SOB) Driven Autotrophic Denitrification.” Water Research 133: 165–172. 29407698 10.1016/j.watres.2018.01.022

[gcb70428-bib-0072] Luo, J. , S. Gu , X. Guo , et al. 2022. “Core Microbiota in the Rhizosphere of Heavy Metal Accumulators and Its Contribution to Plant Performance.” Environmental Science & Technology 56, no. 18: 12975–12987. 36067360 10.1021/acs.est.1c08832

[gcb70428-bib-0073] Magoč, T. , and S. L. Salzberg . 2011. “FLASH: Fast Length Adjustment of Short Reads to Improve Genome Assemblies.” Bioinformatics 27, no. 21: 2957–2963. 21903629 10.1093/bioinformatics/btr507 PMC3198573

[gcb70428-bib-0074] Mahal, N. K. , J. E. Sawyer , J. Iqbal , A. M. Sassman , R. Mathur , and M. J. Castellano . 2022. “Role of Sulfur Mineralization and Fertilizer Source in Corn and Soybean Production Systems.” Soil Science Society of America Journal 86, no. 4: 1058–1071.

[gcb70428-bib-0075] Majumdar, D. , H. Pathak , S. Kumar , and M. Jain . 2002. “Nitrous Oxide Emission From a Sandy Loam Inceptisol Under Irrigated Wheat in India as Influenced by Different Nitrification Inhibitors.” Agriculture, Ecosystems & Environment 91, no. 1–3: 283–293.

[gcb70428-bib-0076] Marietou, A. 2016. “Nitrate Reduction in Sulfate‐Reducing Bacteria.” FEMS Microbiology Letters 363, no. 15: fnw155. 10.1093/femsle/fnw155. 27364687

[gcb70428-bib-0077] Marietou, A. , L. Griffiths , and J. Cole . 2009. “Preferential Reduction of the Thermodynamically Less Favorable Electron Acceptor, Sulfate, by a Nitrate‐Reducing Strain of the Sulfate‐Reducing Bacterium Desulfovibrio Desulfuricans 27774.” Journal of Bacteriology 191, no. 3: 882–889. 19047345 10.1128/JB.01171-08 PMC2632061

[gcb70428-bib-0078] Menard, V. , and G. P. Demopoulos . 2007. “Gas Transfer Kinetics and Redox Potential Considerations in Oxidative Precipitation of Manganese From an Industrial Zinc Sulphate Solution With SO_2_/O_2_ .” Hydrometallurgy 89, no. 3–4: 357–368.

[gcb70428-bib-0079] Meulepas, R. J. , A. J. Stams , and P. N. Lens . 2010. “Biotechnological Aspects of Sulfate Reduction With Methane as Electron Donor.” Reviews in Environmental Science and Biotechnology 9: 59–78.

[gcb70428-bib-0080] Mhuantong, W. , S. Wongwilaiwalin , T. Laothanachareon , et al. 2015. “Survey of Microbial Diversity in Flood Areas During Thailand 2011 Flood Crisis Using High‐Throughput Tagged Amplicon Pyrosequencing.” PLoS One 10, no. 5: e0128043. 26020967 10.1371/journal.pone.0128043 PMC4447364

[gcb70428-bib-0081] Millar, N. , G. P. Robertson , P. R. Grace , R. J. Gehl , and J. P. Hoben . 2010. “Nitrogen Fertilizer Management for Nitrous Oxide (N_2_O) Mitigation in Intensive Corn (Maize) Production: An Emissions Reduction Protocol for US Midwest Agriculture.” Mitigation and Adaptation Strategies for Global Change 15: 185–204.

[gcb70428-bib-0082] Montoya, M. , G. Guardia , J. Recio , et al. 2021. “Zinc‐Nitrogen Co‐Fertilization Influences N_2_O Emissions and Microbial Communities in an Irrigated Maize Field.” Geoderma 383: 114735.

[gcb70428-bib-0083] Mosier, A. , and C. Kroeze . 2000. “Potential Impact on the Global Atmospheric N_2_O Budget of the Increased Nitrogen Input Required to Meet Future Global Food Demands.” Chemosphere ‐ Global Change Science 2, no. 3–4: 465–473.

[gcb70428-bib-0084] Mujakić, I. , P. J. Cabello‐Yeves , C. Villena‐Alemany , et al. 2023. “Multi‐Environment Ecogenomics Analysis of the Cosmopolitan Phylum Gemmatimonadota.” Microbiology Spectrum 11, no. 5: e01112–e01123. 37732776 10.1128/spectrum.01112-23 PMC10581226

[gcb70428-bib-0085] National Institute of Agricultural Sciences . 2022. Fertilization Guidelines by Crop. 5th revised ed [in Korean]. R. D. Administration.

[gcb70428-bib-0086] Ogle, D. H. , J. C. Doll , A. P. Wheeler , and A. Dinno . 2023. “FSA: Simple Fisheries Stock Assessment Methods. R Package Version 0.9, 4.”

[gcb70428-bib-0087] Pan, Y. , L. Ye , and Z. Yuan . 2013. “Effect of H_2_S on N_2_O Reduction and Accumulation During Denitrification by Methanol Utilizing Denitrifiers.” Environmental Science & Technology 47, no. 15: 8408–8415. 23802609 10.1021/es401632r

[gcb70428-bib-0088] Park, Y. L. , H. H. Lee , S. U. Kim , N. Kang , and C. O. Hong . 2022. “Do Metals Increase or Decrease Nitrous Oxide Emissions and Maize Yields From Upland Soils?” Agriculture 12, no. 9: 1458.

[gcb70428-bib-0089] Permyakov, E. A. 2021. “Metal Binding Proteins.” Encyclopedia 1, no. 1: 261–292.

[gcb70428-bib-0090] Philippot, L. , J. Andert , C. M. Jones , D. Bru , and S. Hallin . 2011. “Importance of Denitrifiers Lacking the Genes Encoding the Nitrous Oxide Reductase for N_2_O Emissions From Soil.” Global Change Biology 17, no. 3: 1497–1504.

[gcb70428-bib-0091] Quast, C. , E. Pruesse , P. Yilmaz , et al. 2012. “The SILVA Ribosomal RNA Gene Database Project: Improved Data Processing and Web‐Based Tools.” Nucleic Acids Research 41, no. D1: D590–D596. 23193283 10.1093/nar/gks1219 PMC3531112

[gcb70428-bib-0092] Reay, D. S. , E. A. Davidson , K. A. Smith , et al. 2012. “Global Agriculture and Nitrous Oxide Emissions.” Nature Climate Change 2, no. 6: 410–416.

[gcb70428-bib-0093] Ruser, R. , H. Flessa , R. Russow , G. Schmidt , F. Buegger , and J. Munch . 2006. “Emission of N_2_O, N_2_ and CO_2_ From Soil Fertilized With Nitrate: Effect of Compaction, Soil Moisture and Rewetting.” Soil Biology and Biochemistry 38, no. 2: 263–274.

[gcb70428-bib-0094] Sahrawat, K. 2008. “Soil Fertility Advantages of Submerged Rice Cropping Systems: A Review.” Journal of Sustainable Agriculture 31, no. 3: 5–23.

[gcb70428-bib-0095] Scheer, C. , K. Fuchs , D. E. Pelster , and K. Butterbach‐Bahl . 2020. “Estimating Global Terrestrial Denitrification From Measured N_2_O:(N_2_O+ N_2_) Product Ratios.” Current Opinion in Environmental Sustainability 47: 72–80.

[gcb70428-bib-0096] Schreiber, M. , G. Carey , D. Feinstein , and J. Bahr . 2004. “Mechanisms of Electron Acceptor Utilization: Implications for Simulating Anaerobic Biodegradation.” Journal of Contaminant Hydrology 73, no. 1–4: 99–127. 15336791 10.1016/j.jconhyd.2004.01.004

[gcb70428-bib-0097] Schwertmann, U. , and E. Murad . 1983. “Effect of pH on the Formation of Goethite and Hematite From Ferrihydrite.” Clays and Clay Minerals 31: 277–284.

[gcb70428-bib-0098] Seitz, H.‐J. , and H. Cypionka . 1986. “Chemolithotrophic Growth of *Desulfovibrio desulfuricans* With Hydrogen Coupled to Ammonification of Nitrate or Nitrite.” Archives of Microbiology 146: 63–67.

[gcb70428-bib-0099] Shaaban, M. , Y. Wu , M. S. Khalid , et al. 2018. “Reduction in Soil N_2_O Emissions by pH Manipulation and Enhanced *nosZ* Gene Transcription Under Different Water Regimes.” Environmental Pollution 235: 625–631. 29331895 10.1016/j.envpol.2017.12.066

[gcb70428-bib-0100] Siegbahn, P. E. 2024. “Nitrification Mechanisms for the P460 Enzymes.” Journal of Physical Chemistry B 129, no. 1: 111–116. 39693510 10.1021/acs.jpcb.4c06537 PMC11726666

[gcb70428-bib-0101] Sivan, O. , S. Shusta , and D. Valentine . 2016. “Methanogens Rapidly Transition From Methane Production to Iron Reduction.” Geobiology 14, no. 2: 190–203. 26762691 10.1111/gbi.12172

[gcb70428-bib-0102] Strohm, T. O. , B. Griffin , W. G. Zumft , and B. Schink . 2007. “Growth Yields in Bacterial Denitrification and Nitrate Ammonification.” Applied and Environmental Microbiology 73, no. 5: 1420–1424. 17209072 10.1128/AEM.02508-06 PMC1828769

[gcb70428-bib-0103] Su, J. F. , S. C. Zheng , T. L. Huang , et al. 2016. “Simultaneous Removal of Mn (II) and Nitrate by the Manganese‐Oxidizing Bacterium Acinetobacter sp. SZ28 in Anaerobic Conditions.” Geomicrobiology Journal 33, no. 7: 586–591.

[gcb70428-bib-0104] Syakila, A. , and C. Kroeze . 2011. “The Global Nitrous Oxide Budget Revisited.” Greenhouse Gas Measurement and Management 1, no. 1: 17–26.

[gcb70428-bib-0105] Toyoda, S. , F. Damak , S. Hattori , et al. 2024. “Dynamics of N_2_O Production and Reduction Processes in a Soybean Field Revealed by Isotopocule Analyses.” Soil Biology and Biochemistry 191: 109358.

[gcb70428-bib-0106] Toyoda, S. , T. Kakimoto , K. Kudo , et al. 2021. “Distribution and Production Mechanisms of N_2_O in the Western Arctic Ocean.” Global Biogeochemical Cycles 35, no. 4: e2020GB006881.

[gcb70428-bib-0107] Toyoda, S. , N. Kuroki , N. Yoshida , K. Ishijima , Y. Tohjima , and T. Machida . 2013. “Decadal Time Series of Tropospheric Abundance of N_2_O Isotopomers and Isotopologues in the Northern Hemisphere Obtained by the Long‐Term Observation at Hateruma Island, Japan.” Journal of Geophysical Research: Atmospheres 118, no. 8: 3369–3381.

[gcb70428-bib-0108] Toyoda, S. , S. i. Yamamoto , S. Arai , et al. 2008. “Isotopomeric Characterization of N_2_O Produced, Consumed, and Emitted by Automobiles.” Rapid Communications in Mass Spectrometry 22, no. 5: 603–612. 18247408 10.1002/rcm.3400

[gcb70428-bib-0109] Vandieken, V. , N. Finke , and B. B. Jørgensen . 2006. “Pathways of Carbon Oxidation in an Arctic Fjord Sediment (Svalbard) and Isolation of Psychrophilic and Psychrotolerant Fe (III)‐Reducing Bacteria.” Marine Ecology Progress Series 322: 29–41.

[gcb70428-bib-0110] Voss, M. , N. Choisnard , M. Bartoli , et al. 2024. “3.7—Coastal Nitrogen Cycling—Biogeochemical Processes and the Impacts of Human Activities and Climate Change.” In Treatise on Estuarine and Coastal Science, edited by D. Baird and M. Elliott , Second ed., 225–250. Academic Press. 10.1016/B978-0-323-90798-9.00042-1.

[gcb70428-bib-0111] Wang, C. , B. Amon , K. Schulz , and B. Mehdi . 2021. “Factors That Influence Nitrous Oxide Emissions From Agricultural Soils as Well as Their Representation in Simulation Models: A Review.” Agronomy 11, no. 4: 770.

[gcb70428-bib-0112] Watts, D. , G. Runion , W. Dick , et al. 2023. “Influence of Gypsum and Cover Crop on Greenhouse Gas Emissions in Soybean Cropping Systems.” Journal of Soil and Water Conservation 78, no. 2: 154–162.

[gcb70428-bib-0113] Whelan, J. A. , N. B. Russell , and M. A. Whelan . 2003. “A Method for the Absolute Quantification of cDNA Using Real‐Time PCR.” Journal of Immunological Methods 278, no. 1–2: 261–269. 12957413 10.1016/s0022-1759(03)00223-0

[gcb70428-bib-0114] Worthington, M. J. , R. L. Kucera , I. S. Albuquerque , et al. 2017. “Laying Waste to Mercury: Inexpensive Sorbents Made From Sulfur and Recycled Cooking Oils.” Chemistry—a European Journal 23, no. 64: 16219–16230. 28763123 10.1002/chem.201702871 PMC5724514

[gcb70428-bib-0115] Wrage, N. , G. L. Velthof , H. J. Laanbroek , and O. Oenema . 2004. “Nitrous Oxide Production in Grassland Soils: Assessing the Contribution of Nitrifier Denitrification.” Soil Biology and Biochemistry 36, no. 2: 229–236. 10.1016/j.soilbio.2003.09.009.

[gcb70428-bib-0116] Wu, B. , F. Liu , W. Fang , et al. 2021. “Microbial Sulfur Metabolism and Environmental Implications.” Science of the Total Environment 778: 146085. 33714092 10.1016/j.scitotenv.2021.146085

[gcb70428-bib-0117] Wunder, L. C. , D. A. Aromokeye , X. Yin , et al. 2021. “Iron and Sulfate Reduction Structure Microbial Communities in (Sub‐) Antarctic Sediments.” ISME Journal 15, no. 12: 3587–3604. 34155335 10.1038/s41396-021-01014-9 PMC8630232

[gcb70428-bib-0118] Xin, X. , X. Jiang , J. Su , et al. 2016. “Manganese Oxide Affects Nitrification and Ammonia Oxidizers in Subtropical and Temperate Acid Forest Soils.” Catena 137: 24–30.

[gcb70428-bib-0119] Yang, M. , X. Zhang , S. Ma , et al. 2023. “Shumkonia Mesophila Gen. Nov., sp. Nov., a Novel Representative of Shumkoniaceae Fam. Nov. and Its Potentials for Extracellular Polymeric Substances Formation and Sulfur Metabolism Revealed by Genomic Analysis.” Antonie Van Leeuwenhoek 116, no. 12: 1359–1374. 37843737 10.1007/s10482-023-01878-1

[gcb70428-bib-0120] Yin, Y. , F. Kara‐Murdoch , R. W. Murdoch , et al. 2024. “Nitrous Oxide Inhibition of Methanogenesis Represents an Underappreciated Greenhouse Gas Emission Feedback.” ISME Journal 18, no. 1: wrae027. 10.1093/ismejo/wrae027. 38447133 PMC10960958

[gcb70428-bib-0121] Yu, L. , E. Harris , D. Lewicka‐Szczebak , et al. 2020. “What Can We Learn From N_2_O Isotope Data?–Analytics, Processes and Modelling.” Rapid Communications in Mass Spectrometry 34, no. 20: e8858. 32548934 10.1002/rcm.8858

[gcb70428-bib-0122] Zecchin, S. , J. Wang , M. Martin , M. Romani , B. Planer‐Friedrich , and L. Cavalca . 2023. “Microbial Communities in Paddy Soils: Differences in Abundance and Functionality Between Rhizosphere and Pore Water, the Influence of Different Soil Organic Carbon, Sulfate Fertilization and Cultivation Time, and Contribution to Arsenic Mobility and Speciation.” FEMS Microbiology Ecology 99, no. 11: fiad121. 37804167 10.1093/femsec/fiad121 PMC10630088

[gcb70428-bib-0123] Zhang, J. , W. Zhang , P.‐E. Jansson , and S. O. Petersen . 2022. “Modeling Nitrous Oxide Emissions From Agricultural Soil Incubation Experiments Using CoupModel.” Biogeosciences 19, no. 19: 4811–4832.

[gcb70428-bib-0124] Zhang, Z. , G. Chen , X. Yu , et al. 2024. “A Slow‐Release Fertilizer Containing Cyhalofop‐Butyl Reduces N_2_O Emissions by Slowly Releasing Nitrogen and Down‐Regulating the Relative Abundance of *nirK* .” Science of the Total Environment 906: 167493. 37778565 10.1016/j.scitotenv.2023.167493

[gcb70428-bib-0125] Zhou, H. , J. Long , M. Qin , et al. 2024. “Successful Operation of Nitrifying Granules at Low pH in a Continuous‐Flow Reactor: Nitrification Performance, Granule Stability, and Microbial Community.” Journal of Environmental Management 366: 121793. 38991342 10.1016/j.jenvman.2024.121793

[gcb70428-bib-0126] Zhou, Q. , W. Wu , and J. Wang . 2025. “Simultaneous Occurrence of Sulfate Reduction and Nitrate Reduction in Solid‐Phase Denitrification System.” Chemical Engineering Journal 507: 160570.

