# Supplementary material for: Exploring Sulfate as an Alternative Electron Acceptor: A Potential Strategy to Mitigate N2O Emissions in Upland Arable Soils
Source: Glob Chang Biol. 2025 Aug 13;31(8):e70428. doi: 10.1111/gcb.70428 (PMC12344629; doi:10.1111/gcb.70428)
Supplement: Supplementary file 1 — Data S1: gcb70428‐sup‐0001‐supinfo.zip. [file GCB-31-e70428-s001.zip › gcb70428-sup-0005-TableS1-S6-FigureS1-S8@N2OSO4Upland_Supplementary Tables and Figures.docx]

**Supplementary Information for**

**Exploring Sulfate as an Alternative Electron Acceptor: A Potential Strategy to Mitigate N_2_O Emissions in Upland Arable Soils**

Content of the file: Supplementary tables 1, 2, 3, 4, 5 and 6, supplementary figures 1, 2, 3, 4, 5, 6, 7, and 8.

**Supplementary Table 1. Soil characteristics of the studied soil for microcosm and field-based manipulation experiments**

| Items | Value |
| --- | --- |
| Particle size distribution  Sand (%)  Silt (%)  Clay (%)  Soil texture  Bulk density (g cm^−1^)  pH (1:5 with H_2_O)  Organic matter (g kg^−1^)  Total nitrogen (g kg^−1^)  Available P_2_O_5_ (mg kg^−1^)  Inorganic nitrogen  NH_4_^+^  NO_3_^−^  Extractable SO_4_^2−^ (mg kg^−1^)  Exchangeable cations  K (cmol_c_ kg^−1^)  Ca (cmol_c_ kg^−1^)  Mg (cmol_c_ kg^−1^)  Cation exchangeable capacity (cmol_c_ kg^−1^) | 59.4  25.7  14.9  Sandy clay loam  1.31  6.40  17.6  1.05  142  13.8  10.7  8.78  0.42  4.76  1.02  6.85 |

**Supplementary Table 2. Information on targeting primers**

| **Primer** | **Sequence (5′ → 3′)** | **Annealing T.** | **Size (bp)** | **PCR efficiency** | **Reference** |
| --- | --- | --- | --- | --- | --- |
| Bacterial 16S rRNA―341F | CGGCAACGAGCGCAACCC | 50ºC | 193 | 93.2% | (Denman & McSweeney, 2006) |
| Bacterial 16S rRNA―797R | CCATTGTAGCACGTGTGTAGCC |  |  |  |  |
| *amo*A-1F | GGGGTTTCTACTGGTGGT | 60ºC | 491 | 96.7% | (Rotthauwe et al., 1997) |
| *amo*A-2R | CCCCTCKGSAAAGCCTTCTTC |  |  |  |  |
| *hao*-1F | TGCGTGGAAGTGCTCAC | 50ºC | 992 | 89.8% | (Schmid et al., 2008) |
| *hao*-3R | AGAGTAAGGAGTCTCGGGCAAA |  |  |  |  |
| *nir*K-1F | GGRATGGTKCCSTGGCA | 65ºC – 60ºC | 514 | 107.3% | (López-Gutiérrez et al., 2004) |
| *nir*K-5R | GCCTCGATCAGRTTRTGG |  |  |  |  |
| *nir*S-1F | CCTAYTGGCCGCCRCART | 65ºC – 60ºC | 890 | 111.0% | (Braker et al., 1998) |
| *nir*S-6R | CGTTGAACTTRCCGGT |  |  |  |  |
| *cnor*B-2F | GACAAGNNNTACTGGTGGT | 55ºC | 389 | 103.6% | (Braker & Tiedje, 2003) |
| *cnor*B-6R | GAANCCCCANACNCCNGC |  |  |  |  |
| *nos*Z-1F | WCSYTGTTCMTCGACAGCCAG | 65ºC – 60ºC | 700 | 95.5% | (Henry et al., 2006) |
| *nos*Z-2R | CAKRTGCAKSGCRTGGCAGAA |  |  |  |  |
| *DSRp2060F* | CCAACATCGTYCAYACCCAGGG | 55ºC | 350 | 101.9% | (Miletto et al., 2007) |
| *DSR4R* | GTGTAGCAGTTACCGCA |  |  |  |  |

**Supplementary Table 3. DADA2 denoising statistics of 16S rRNA gene amplicon sequence variants among treatments**

| Treatment | No. of input paired reads | Merged reads^1^ | Quality filtered reads | Denoised reads | Chimera-filtered reads | Taxa-filtered reads^2^ |
| --- | --- | --- | --- | --- | --- | --- |
| CON | 61,072 | 57,028 | 46,312 | 41,584 | 41,157 | 41,125 |
| CON | 61,279 | 56,905 | 45,880 | 40,434 | 39,865 | 39,824 |
| CON | 58,338 | 55,104 | 45,378 | 41,258 | 40,782 | 40,746 |
| MS | 67,952 | 64,665 | 50,117 | 45,267 | 44,729 | 44,638 |
| MS | 46,418 | 43,860 | 43,355 | 38,786 | 38,447 | 38,378 |
| MS | 61,582 | 57,748 | 53,201 | 47,556 | 46,946 | 46,862 |
| ZVM | 65,179 | 61,444 | 53,815 | 48,766 | 47,911 | 47,851 |
| ZVM | 55,552 | 52,649 | 35,708 | 31,479 | 31,154 | 31,118 |
| ZVM | 68,042 | 64,525 | 47,497 | 42,534 | 41,957 | 41,872 |

^1^Forward and reverse reads were merged using FLASH2

^2^Amplicon sequence variants identified as unassigned, mitochondria, chloroplast were filtered out

**Supplementary Table 4. Analysis of compositions of microbiomes with bias correction 2 (ANCOME-BC2)**

(Attached excel file)

| Metal sulfates | Nitrogen uptake by crops (kg ha^−1^) | | | | | | |
| --- | --- | --- | --- | --- | --- | --- | --- |
|  | Year 1 (2019) | | | | Year 2 (2020) | | |
|  | Grain | Stem and leaf | Total |  | Grain | Stem and leaf | Total |
| Control | 8.99 (1.40) | 0.72 (0.11) | 9.71 (1.31) |  | 7.93 (1.12) | 0.75 (0.07) | 8.68 (1.18) |
| Iron sulfate | 8.73 (1.70) | 0.82 (0.17) | 9.55 (1.73) |  | 8.28 (1.33) | 0.75 (0.12) | 9.03 (1.46) |
| Manganese sulfate | 8.43 (1.08) | 0.97 (0.22) | 9.40 (1.25) |  | 7.99 (0.52) | 0.80 (0.52) | 8.78 (0.58) |
| Zinc sulfate | 8.33 (0.53) | 0.92 (0.20) | 9.25 (0.57) |  | 7.13 (1.61) | 1.01 (0.36) | 8.14 (1.31) |

**Supplementary Table 5. Nitrogen uptake by maize crops in grain, stem, and leaf, and total biomass under different metal sulfate treatments (Control, Iron sulfate, Manganese sulfate, and Zinc sulfate) during the two-year field experiment (Year 1 and Year 2).** Values represent the mean and standard deviation (in parentheses). No statistically significant differences were observed among treatments.

**Supplementary Table 6. Summary of studies on the effects of sulfate addition on N_2_O emissions based on systematic literature review (PRISMA).** The table presents details on sulfate materials, application rates, experimental types, plant species, and N_2_O emissions under both conventional and sulfate-treated conditions. The percentage change in N_2_O emissions response by sulfate addition is indicated in the “Changes in sulfate (%)” column. References are provided for each study to share the original source of the data.

| Sulfate materials | | Sulfate addition rates | Plant Species | N_2_O emissions | | | | | | | References |
| --- | --- | --- | --- | --- | --- | --- | --- | --- | --- | --- | --- |
|  |  |  |  | Convention | | Treatment | | Unit | Changes in Sulfate (%) | |  |
| Ammonium sulfate | | 516 kg ha^−1^ | Carrot | 536 | 219 | | | g N ha^−1^ | −59 | | (Li et al., 2002) |
|  |  | 1031 kg ha^−1^ |  | 722 | 316 | | |  | −56 | |  |
| Ammonium sulfate | | 103 kg ha^−1^ | Lettuce | 0.21 | 0.24 | | | kg N ha^−1^ | 14 | | (Hoang Thi Thai Hoa et al., 2018) |
|  |  |  |  | 0.3 | 0.24 | | |  | −20 | |  |
|  |  |  |  | 0.17 | 0.18 | | |  | 6 | |  |
|  |  |  |  | 0.25 | 0.18 | | |  | −28 | |  |
|  |  | 206 kg ha^−1^ |  | 0.38 | 0.51 | | |  | 34 | |  |
|  |  |  |  | 0.59 | 0.51 | | |  | −14 | |  |
|  |  |  |  | 0.29 | 0.29 | | |  | 0 | |  |
|  |  |  |  | 0.45 | 0.29 | | |  | −36 | |  |
|  |  | 310 kg ha^−1^ |  | 0.64 | 0.89 | | |  | 39 | |  |
|  |  |  |  | 1.01 | 0.89 | | |  | −12 | |  |
|  |  |  |  | 0.42 | 0.37 | | |  | −12 | |  |
|  |  |  |  | 0.72 | 0.37 | | |  | −49 | |  |
| Ammonium sulfate | | 550 kg ha^−1^ | Sugarcane | 1143 | 951 | | | g N ha^−1^ | −17 | | (Weier, 1999) |
| Ammonium sulfate | | 121 kg ha^−1^ | Eucalyptus urograndis | 1010 | 623 | | | mg N m^−2^ | −38 | | (Ibarr et al., 2021) |
| Ammonium sulfate | | 275 kg ha^−1^ | Maize | 81 | 113 | | | mg N_2_O m^−2^ | 40 | | (Martins et al., 2015) |
| Ammonium sulfate | | 516 kg ha^−1^ | Maize | 1.71 | 1.65 | | | kg N ha^−1^ | −4 | | (Gu et al., 2016) |
| Ammonium sulfate | | 138 kg ha^−1^ | Marandu palisade grass | 16.3 | 14.4 | | | μg N m^−2^ | −12 | | (do Nascimento et al., 2021) |
|  |  | 275 kg ha^−1^ |  | 36.5 | 21.7 | | |  | −41 | |  |
| Ammonium sulfate | | 516 kg ha^−1^ | Corn | 0.48 | 0.68 | | | kg N ha^−1^ | 42 | | (Zanatta et al., 2010) |
| Ammonium sulfate | | 347 kg ha^−1^ | Cotton | 1617 | 2228 | | | g N ha^−1^ | 38 | | (Watts et al., 2023) |
|  |  |  |  | 1617 | 986 | | |  | −39 | |  |
|  |  |  |  | 1313 | 1708 | | |  | 30 | |  |
|  |  |  |  | 1313 | 1054 | | |  | −20 | |  |
|  |  |  |  | 1946 | 1806 | | |  | −7 | |  |
|  |  |  |  | 1946 | 1100 | | |  | −43 | |  |
| Zinc sulfate | | 10 kg Zn ha^−1^ | Maize | 0.12 | 0.16 | | | kg N ha^−1^ | 33 | | (Montoya et al., 2021) |
|  |  |  |  | 0.80 | 1.56 | | |  | 95 | |  |
| Zinc sulfate | | 10 kg Zn ha^−1^ | Winter wheat | 232 | 241 | | | g N ha^−1^ | 4 | | (Montoya et al., 2018) |
| Ammonium sulfate | | 550 kg ha^−1^ | Winter wheat | 14.1 | 26.9 | | | mg N dm^−3^ | 91 | | (Debreczeni & and Berecz, 1998) |
| Ammonium sulfate | | 757 kg ha^−1^ | Winter wheat | 521 | 367 | | | g N ha^−1^ | −29 | | (Lebender et al., 2014) |
|  |  |  |  | 617 | 554 | | |  | −10 | |  |
|  |  |  |  | 394 | 461 | | |  | 17 | |  |
| Ammonium sulfate | | 516 kg ha^−1^ | Wheat | 36.9 | 33.2 | | | kg N ha^−1^ | −10 | | (Kumar & Bordoloi, 2024) |
| Gypsum  (CaSO_4_) | | 2Mg ha^−1^ | Soybean-Maize | 0.69 | 0.66 | | | kg N ha^−1^ | −5 | | (Barcelos et al., 2022) |
| Gypsum  (CaSO_4_) | | 2200 kg ha^−1^ | Soybean | 319 | 374 | | | g ha^−1^ yr^−1^ | 17 | | (Watts et al., 2023) |
|  |  |  |  | 343 | 317 | | |  | −8 | |  |
|  |  |  |  | 621 | 527 | | |  | −15 | |  |
|  |  |  |  | 200 | 244 | | |  | 22 | |  |
|  |  |  |  | 317 | 276 | | |  | −13 | |  |
|  |  |  |  | 313 | 482 | | |  | 54 | |  |
|  |  |  |  | 1145 | 925 | | |  | −19 | |  |
|  |  |  |  | 500 | 581 | | |  | 16 | |  |
|  |  |  |  | 1367 | 1262 | | |  | −8 | |  |
|  |  |  |  | 299 | 359 | | |  | 20 | |  |
|  |  |  |  | 1382 | 1392 | | |  | 1 | |  |
|  |  |  |  | 428 | 467 | | |  | 9 | |  |
|  |  |  |  | 712 | 809 | | |  | 14 | |  |
| Gypsum  (CaSO_4_) | | 2200 kg ha^−1^ | Corn-Soybean | 492 | 499 | | | g ha^−1^ yr^−1^ | 1 | | (Watts et al., 2023) |
|  |  |  |  | 509 | 611 | | |  | 20 | |  |
|  |  |  |  | 301 | 344 | | |  | 14 | |  |
|  |  |  |  | 406 | 329 | | |  | −19 | |  |
|  |  |  |  | 2783 | 1905 | | |  | −32 | |  |
|  |  |  |  | 3213 | 2731 | | |  | −15 | |  |
|  |  |  |  | 280 | 215 | | |  | −23 | |  |
|  |  |  |  | 493 | 472 | | |  | −4 | |  |
| CaSO_4_ | | 5 g kg^−1^ | − | 232 | 201 | | | μg kg^−1^ | −13 | | (Ribeiro et al., 2024) |
|  |  |  |  | 82 | 158 | | |  | 92 | |  |
|  |  |  |  | 85 | 41 | | |  | −52 | |  |
|  |  |  |  | 9 | 8 | | |  | −7 | |  |
| Gypsum  (CaSO_4_) | 0.72 g kg^−1^ | | − | 12 | | 11 | mg m^−2^ yr^−1^ | | −8 | (Gao et al., 2024) | |
|  |  |  |  | 19 | | 17 |  |  | −11 |  |  |
| Thiosulphate | 18 kg ha^−1^ | | Wheat | 1.43 | | 1.19 | kg ha^−1^ | | −17 | (Majumdar et al., 2002) | |
| Na_2_SO_4_ | 45 kg ha^−1^ | | Pine | 0.85 | | 0.62 | kg ha^−1^ | | −27 | (Fan et al., 2017) | |
|  |  |  |  | 1.08 | | 0.80 |  |  | −26 |  |  |
|  |  |  |  | 0.45 | | 0.38 |  |  | −16 |  |  |
|  |  |  |  | 1.72 | | 1.06 |  |  | −38 |  |  |
|  |  |  |  | 1.89 | | 1.24 |  |  | −34 |  |  |
|  |  |  |  | 0.39 | | 0.33 |  |  | −15 |  |  |

**Supplementary Fig. 1. Daily variations in water-filled pore space (WFPS, % *v/v*), air temperature (℃), and precipitation (mm) during Year 1 (2018) and Year 2 (2019) of the field experiment.** WFPS is represented by the black line (right y-axis), air temperature by red dots (left y-axis), and precipitation by blue bars (secondary right y-axis). The dotted vertical line indicates the transition between the two calendar years.


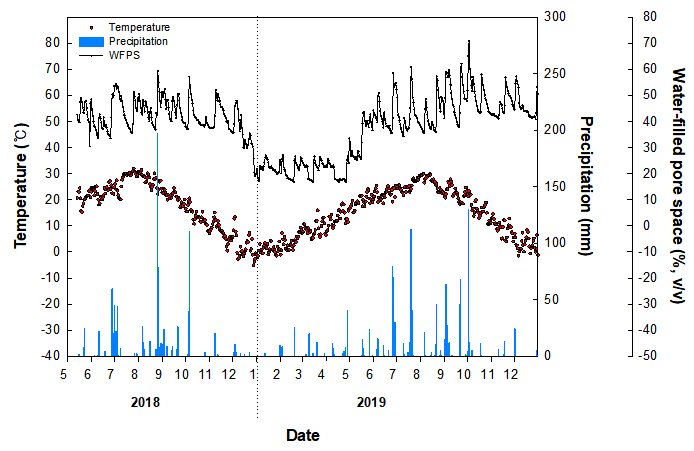


**Supplementary Fig. 2. N_2_O emissions at time-batch microcosm experiments.** Daily N_2_O emissions comparison for (a) metal sulfates (control, FeSO_4_, MnSO_4_, and ZnSO_4_) and (b) zero-valent metals (control, Fe^0^, Mn^0^, and Zn^0^) from incubated soils for 7, 14, and 21 days. Statistically significant differences among the treatment groups based on one-way ANOVA by the least significant difference test (*p*<0.05) are indicated by differing letters.

**
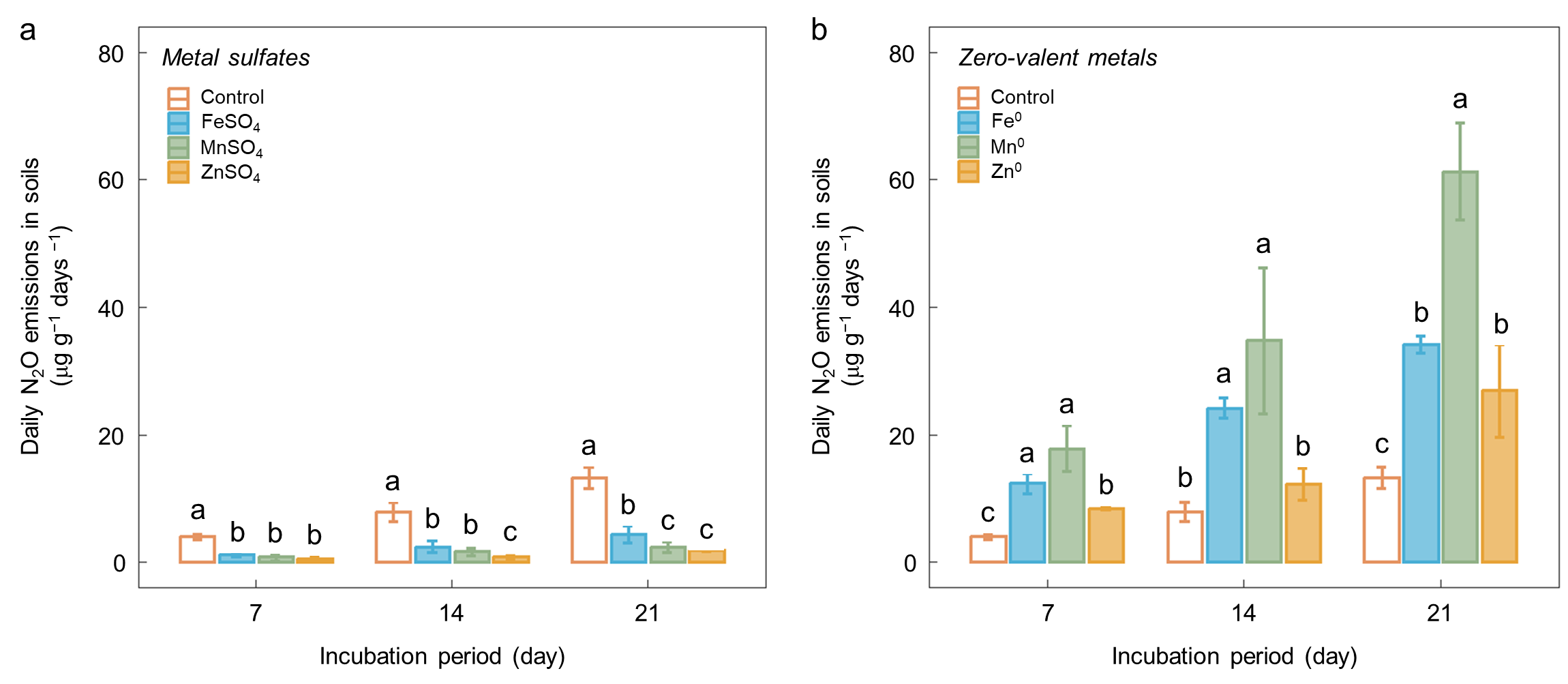
**

**Supplementary Fig. 3. Rarefaction curves of alpha diversity indices.** ASV, amplicon sequence variants; CON, control; MS, metal sulfate; AVM, zero-valent metal.


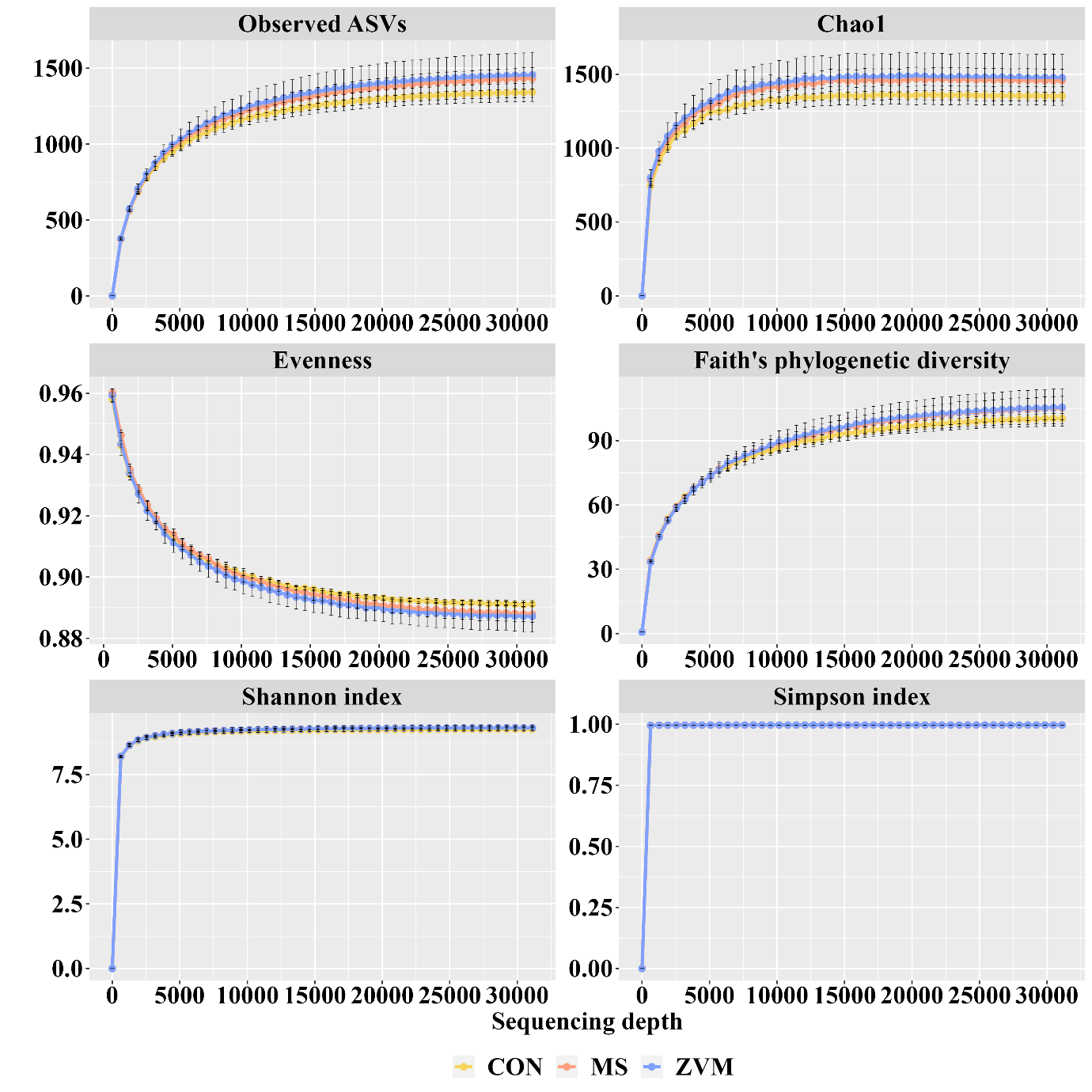


**Supplementary Fig. 4. Differences in alpha diversity measurements of the soil microbiota in treatment groups** (CON, control; MS, metal sulfate; ZVM, zero-valent metal). Boxplots represent six diversity indices: Observed amplicon sequence variants (ASVs), Chao1, Evenness, Faith’s phylogenetic diversity, Shannon index, and Simpson index. Triangles within boxes indicate group means. *p*-values are based on one-way ANOVA to test significant differences among treatments. No statistically significant differences were found across treatments (*p*>0.05).

**
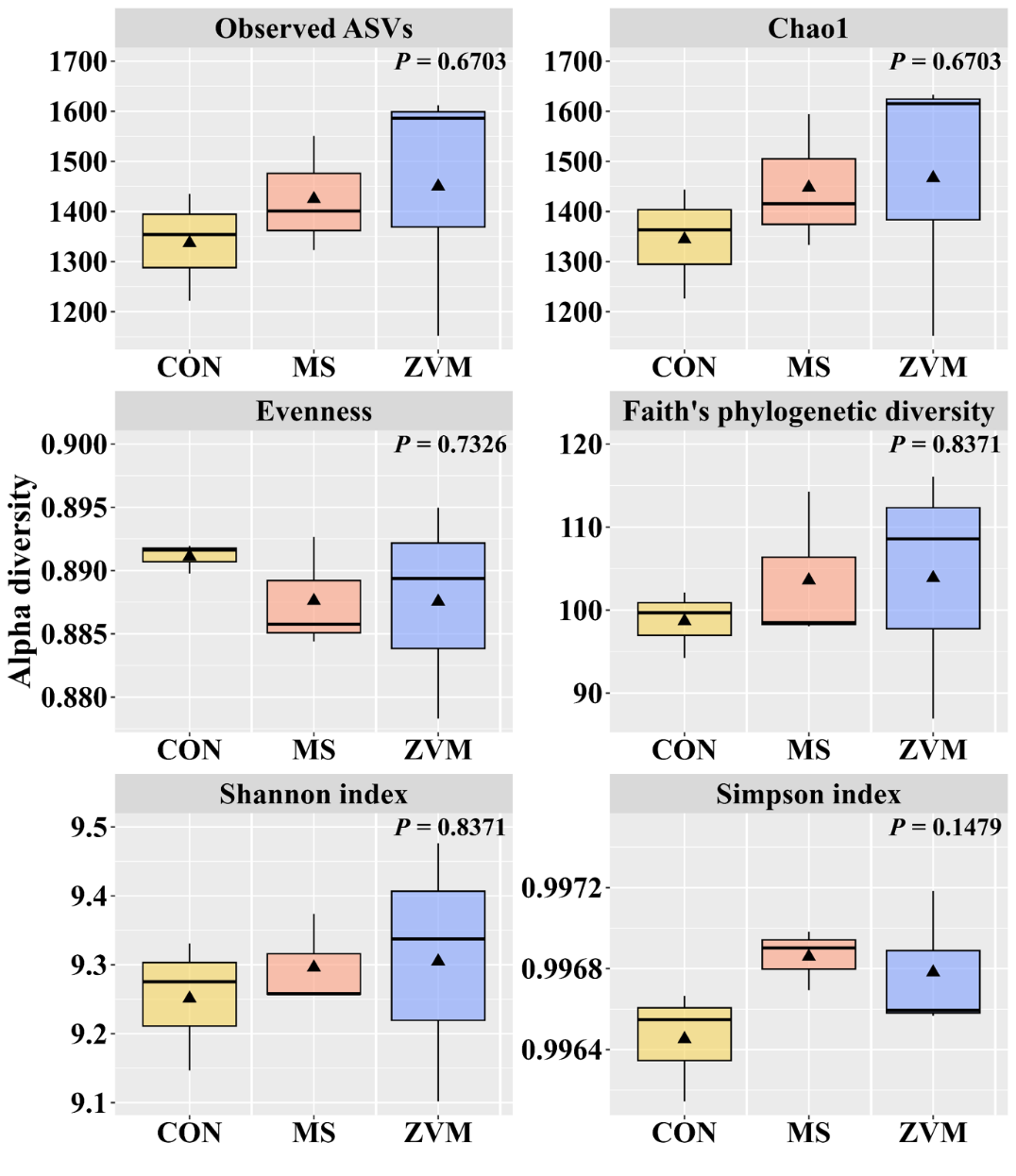
**

**Supplementary Fig. 5. Daily N_2_O fluxes with metal-sulfates from May 2020 through December 2021.** The flux measurements were conducted across two growing seasons (Year 1 and 2), with cultivation periods indicated by shaded blue areas. Error bars represent the standard deviation of the mean values (*n* = 3).

**
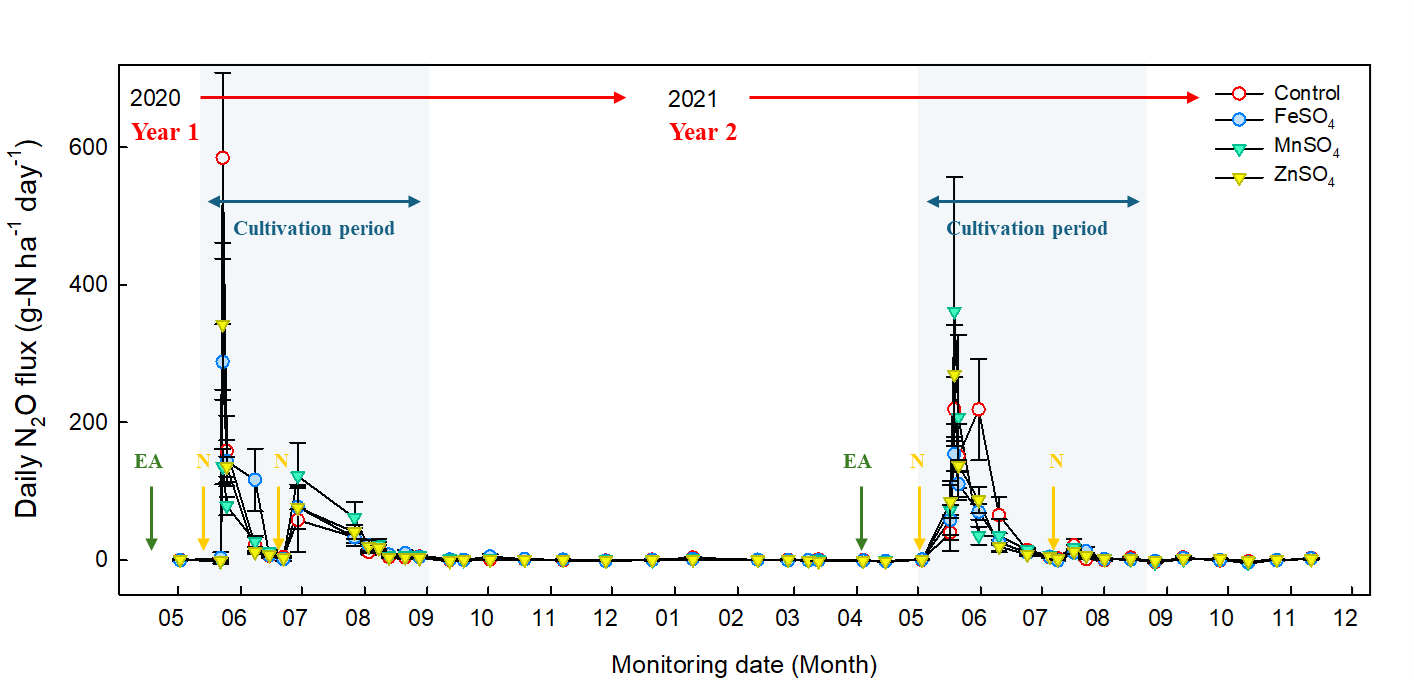
**

**Supplementary Fig. 6. Dynamics of inorganic nitrogen concentrations in response to metal sulfate application during the 21-day incubation.** (a) Ammonium (NH_4_^+^) and (b) nitrate (NO_3_^−^) concentration was measured at days 7, 14, and 21 under different treatments (Control, FeSO_4_, MnSO_4_, and ZnSO_4_). Erro bars represent standard error (*n* = 3). NS indicates no significant differences among treatments at a given time point (*p*>0.05). Different lowercase letters denote statistically significant differences among treatments at each time point based on one-way ANOVA followed by the least significant difference (LSD) test (*p*<0.05).

**
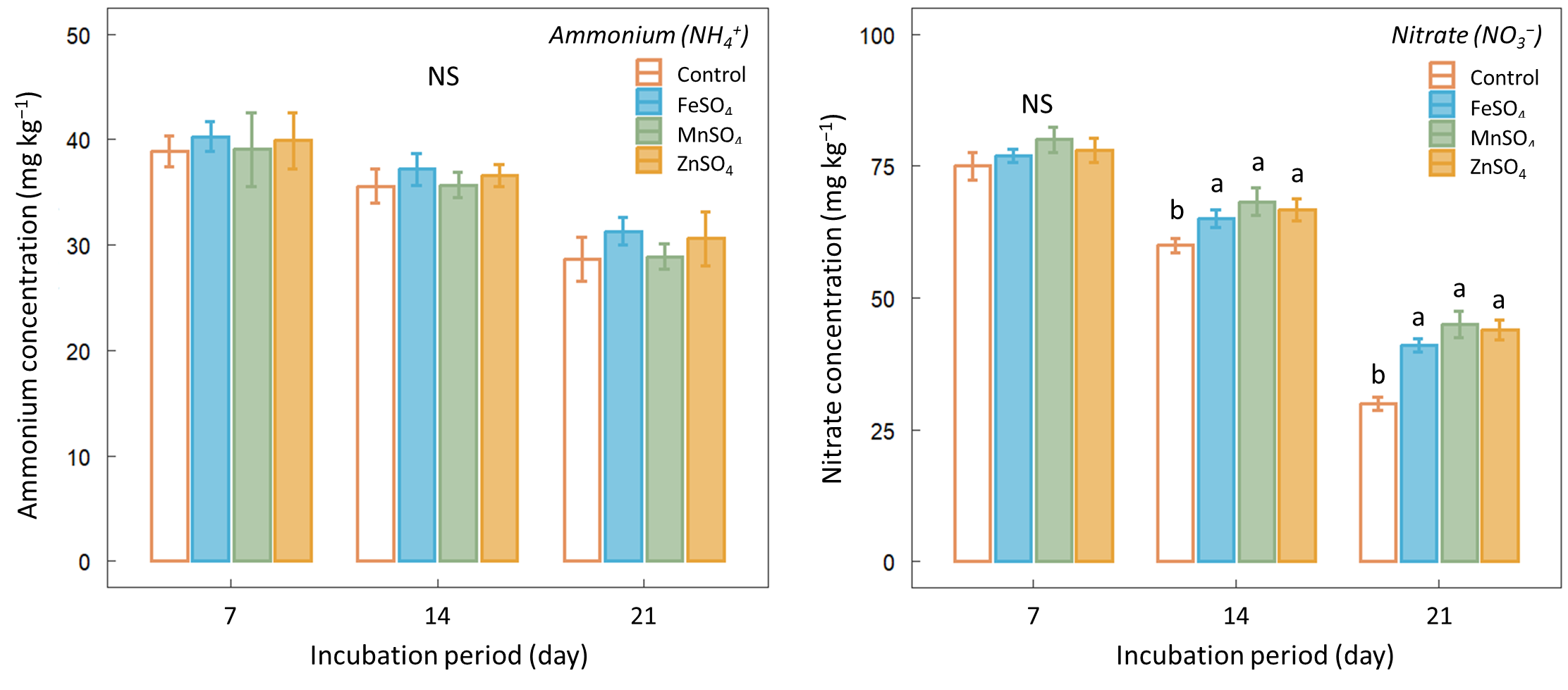
**

**Supplementary Fig. 7. Activity of sulfate reducing bacteria in soils with treatments.** The transcript abundance of *dsrA* gene per gram of dry soil treated with metal sulfates (FeSO_4_, MnSO_4_, and ZnSO_4_) and zero-valent metals (Fe^0^, Mn^0^, and Zn^0^) after 21 days of incubation. The thick central line represents the median value, the boxed areas represent the interquartile range, and the whiskers show the maximum and minimum values (*N* = 6). Statistically significant differences among the treatment groups, determined by one-way ANOVA with the least significant difference test (*p*<0.05), are indicated by differing letters.

**
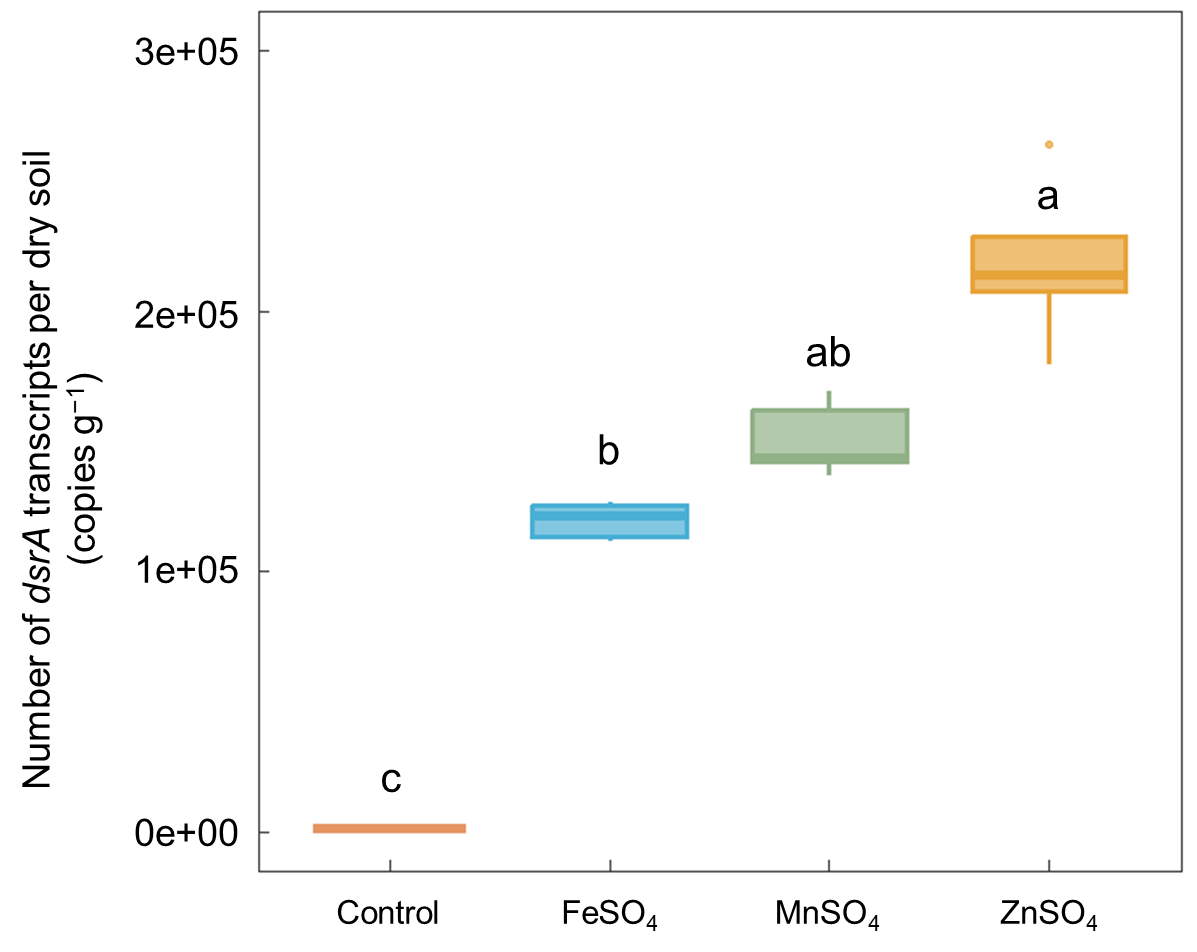
**

**Supplementary Fig. 8. Chanes in soil pH at three key stages of the cropping period (transplanting, cultivating, and harvest) during the second year of the field experiment.** Soil pH was measured in four replicates (*n* = 4) for each treatment: control, FeSO_4_, MnSO_4_, and ZnSO_4_. Error bars represent standard deviation. NS indicates no statistically significant differences among treatments at each time point (*p*>0.05).

**
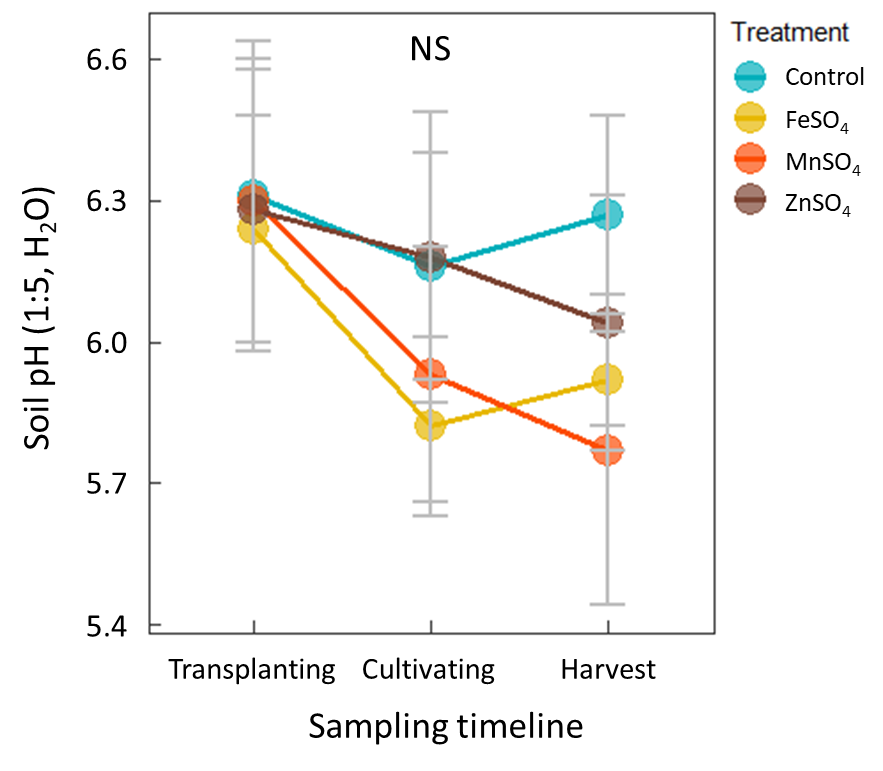
**

**References**

Barcelos, J. P. d. Q., de Souza, M., Nascimento, C. A. C. d., & Rosolem, C. A. (2022). Soil acidity amelioration improves N and C cycles in the short term in a system with soybean followed by maize-guinea grass intercropping. *Geoderma*, *421*, 115909. <https://doi.org/https://doi.org/10.1016/j.geoderma.2022.115909>

Braker, G., Fesefeldt, A., & Witzel, K.-P. (1998). Development of PCR Primer Systems for Amplification of Nitrite Reductase Genes (*nirK* and *nirS*) To Detect Denitrifying Bacteria in Environmental Samples. *Applied and environmental microbiology*, *64*(10), 3769-3775. <https://doi.org/doi:10.1128/AEM.64.10.3769-3775.1998>

Braker, G., & Tiedje, J. M. (2003). Nitric Oxide Reductase (*norB*) Genes from Pure Cultures and Environmental Samples. *Applied and environmental microbiology*, *69*(6), 3476-3483. <https://doi.org/doi:10.1128/AEM.69.6.3476-3483.2003>

Debreczeni, K., & and Berecz, K. (1998). Monitoring of gaseous nitrogen losses from nitrogen fertilizers in model experiments. *Communications in Soil Science and Plant Analysis*, *29*(11-14), 2207-2216. <https://doi.org/10.1080/00103629809370103>

Denman, S. E., & McSweeney, C. S. (2006). Development of a real-time PCR assay for monitoring anaerobic fungal and cellulolytic bacterial populations within the rumen. *FEMS Microbiology Ecology*, *58*(3), 572-582. <https://doi.org/10.1111/j.1574-6941.2006.00190.x>

do Nascimento, A. F., de Oliveira, C. M., Pedreira, B. C., Pereira, D. H., & Rodrigues, R. R. d. A. (2021). Nitrous oxide emissions and forage accumulation in the Brazilian Amazon forage-livestock systems submitted to N input strategies. *Grassland Science*, *67*(1), 63-72. <https://doi.org/https://doi.org/10.1111/grs.12287>

Fan, J., Xu, Y., Chen, Z., Xiao, J., Liu, D., Luo, J., Bolan, N., & Ding, W. (2017). Sulfur deposition suppressed nitrogen-induced soil N_2_O emission from a subtropical forestland in southeastern China. *Agricultural and Forest Meteorology*, *233*, 163-170.

Gao, P., Yan, X., Xia, X., Liu, D., Guo, S., Ma, R., Lou, Y., Yang, Z., Wang, H., & Yang, Q. (2024). Effects of the three amendments on NH_3_ volatilization, N_2_O emissions, and nitrification at four salinity levels: An indoor experiment. *Journal of Environmental Management*, *354*, 120399.

Gu, J., Xiang, H., Kuang, F., Hao, Y., Qu, D., & Zhu, B. (2016). Simulating denitrification and nitrous oxide emissions from subtropical maize-winter wheat rotations in Southwestern China using NOEv2 model. *Agriculture, ecosystems & environment*, *230*, 127-138. <https://doi.org/https://doi.org/10.1016/j.agee.2016.05.034>

Henry, S., Bru, D., Stres, B., Hallet, S., & Philippot, L. (2006). Quantitative Detection of the *nosZ* Gene, Encoding Nitrous Oxide Reductase, and Comparison of the Abundances of 16S rRNA, *narG*, *nirK*, and *nosZ* Genes in Soils. *Applied and environmental microbiology*, *72*(8), 5181-5189. <https://doi.org/doi:10.1128/AEM.00231-06>

Hoang Thi Thai Hoa, H. T. T. H., Do Dinh Thuc, D. D. T., & Trinh Thi Sen, T. T. S. (2018). Nitrogen fertilization management and nitrous oxide emission in lettuce vegetable fields in Central Vietnam. *International Journal of Agriculture and Biology*, *20*(2), 249–254. <https://doi.org/10.17957/ijab/15.0484>

Ibarr, M. A., Zanatta, J. A., Dieckow, J., Ribeiro, R. H., Rachwal, M. F. G., & Stahl, J. (2021). Nitrous oxide and methane emissions from soil and nitrogen uptake by eucalyptus fertilized with enhanced efficiency fertilizers. *Plant and Soil*, *463*(1), 615-630. <https://doi.org/10.1007/s11104-021-04938-5>

Kumar, R., & Bordoloi, N. (2024). Combined impact of reduced N fertilizer and green manure on wheat yield, nitrogen use efficiency and nitrous oxide (N_2_O) emissions reduction in Jharkhand, eastern India. *Field Crops Research*, *318*, 109591. <https://doi.org/https://doi.org/10.1016/j.fcr.2024.109591>

Lebender, U., Senbayram, M., Lammel, J., & Kuhlmann, H. (2014). Effect of mineral nitrogen fertilizer forms on N₂O emissions from arable soils in winter wheat production. *Journal of Plant Nutrition and Soil Science*, *177*(5), 722-732. <https://doi.org/10.1002/jpln.201300326>

Li, X., Inubushi, K., & Sakamoto, K. (2002). Nitrous oxide concentrations in an Andisol profile and emissions to the atmosphere as influenced by the application of nitrogen fertilizers and manure. *Biology and Fertility of Soils*, *35*(2), 108-113. <https://doi.org/10.1007/s00374-002-0447-7>

López-Gutiérrez, J. C., Henry, S., Hallet, S., Martin-Laurent, F., Catroux, G., & Philippot, L. (2004). Quantification of a novel group of nitrate-reducing bacteria in the environment by real-time PCR. *Journal of Microbiological Methods*, *57*(3), 399-407. <https://doi.org/https://doi.org/10.1016/j.mimet.2004.02.009>

Majumdar, D., Pathak, H., Kumar, S., & Jain, M. (2002). Nitrous oxide emission from a sandy loam Inceptisol under irrigated wheat in India as influenced by different nitrification inhibitors. *Agriculture, ecosystems & environment*, *91*(1-3), 283-293.

Martins, M. R., Jantalia, C. P., Polidoro, J. C., Batista, J. N., Alves, B. J. R., Boddey, R. M., & Urquiaga, S. (2015). Nitrous oxide and ammonia emissions from N fertilization of maize crop under no-till in a Cerrado soil. *Soil and Tillage Research*, *151*, 75-81. <https://doi.org/https://doi.org/10.1016/j.still.2015.03.004>

Miletto, M., Bodelier, P. L. E., & Laanbroek, H. J. (2007). Improved PCR-DGGE for high resolution diversity screening of complex sulfate-reducing prokaryotic communities in soils and sediments. *Journal of Microbiological Methods*, *70*(1), 103-111. <https://doi.org/https://doi.org/10.1016/j.mimet.2007.03.015>

Montoya, M., Castellano-Hinojosa, A., Vallejo, A., Álvarez, J. M., Bedmar, E. J., Recio, J., & Guardia, G. (2018). Zinc fertilizers influence greenhouse gas emissions and nitrifying and denitrifying communities in a non-irrigated arable cropland. *Geoderma*, *325*, 208-217. <https://doi.org/https://doi.org/10.1016/j.geoderma.2018.03.035>

Montoya, M., Guardia, G., Recio, J., Castellano-Hinojosa, A., Ginés, C., Bedmar, E. J., Álvarez, J. M., & Vallejo, A. (2021). Zinc-nitrogen co-fertilization influences N_2_O emissions and microbial communities in an irrigated maize field. *Geoderma*, *383*, 114735. <https://doi.org/https://doi.org/10.1016/j.geoderma.2020.114735>

Ribeiro, P. L., Hamta, Z., & Mühling, K. H. (2024). Greenhouse gas emissions from sandy soils amended with lime and gypsum under different initial soil pH and nitrogen sources. *Soil Use and Management*, *40*(3), e13089.

Rotthauwe, J. H., Witzel, K. P., & Liesack, W. (1997). The ammonia monooxygenase structural gene *amoA* as a functional marker: molecular fine-scale analysis of natural ammonia-oxidizing populations. *Applied and environmental microbiology*, *63*(12), 4704-4712. <https://doi.org/doi:10.1128/aem.63.12.4704-4712.1997>

Schmid, M. C., Hooper, A. B., Klotz, M. G., Woebken, D., Lam, P., Kuypers, M. M. M., Pommerening-Roeser, A., Op Den Camp, H. J. M., & Jetten, M. S. M. (2008). Environmental detection of octahaem cytochrome c hydroxylamine/hydrazine oxidoreductase genes of aerobic and anaerobic ammonium-oxidizing bacteria. *Environmental Microbiology*, *10*(11), 3140-3149. <https://doi.org/https://doi.org/10.1111/j.1462-2920.2008.01732.x>

Watts, D., Runion, G., Dick, W., Gonzalez, J., Islam, K., Flanagan, D., Fausey, N., VanToai, T., Batte, M., & Reeder, R. (2023). Influence of gypsum and cover crop on greenhouse gas emissions in soybean cropping systems. *Journal of Soil and Water Conservation*, *78*(2), 154-162.

Weier, K. L. (1999). N_2_O and CH_4_ emission and CH_4_ consumption in a sugarcane soil after variation in nitrogen and water application. *Soil Biology and Biochemistry*, *31*(14), 1931-1941. <https://doi.org/https://doi.org/10.1016/S0038-0717(99)00111-X>

Zanatta, J. A., Bayer, C., Vieira, F. C., Gomes, J., & Tomazi, M. (2010). Nitrous oxide and methane fluxes in South Brazilian Gleysol as affected by nitrogen fertilizers. *Revista Brasileira de Ciência do Solo*, *34*, 1653-1665. <https://www.scielo.br/j/rbcs/a/6f6fpVdY9MvWZzWpqTdgt3D/?lang=en>
